# 3'UTR of tobacco vein mottling virus regulates downstream GFP expression and changes in host gene expression

**DOI:** 10.3389/fmicb.2024.1477074

**Published:** 2024-10-14

**Authors:** Zhenqi Sun, Dongyang Liu, Bin Li, Fangfang Yan, Yuhu Wang, Tianqi Yang, Haijuan Wang, Jiaxin Xu, Hongyou Zhou, Mingmin Zhao

**Affiliations:** ^1^College of Horticulture and Plant Protection, Inner Mongolia Agricultural University, Hohhot, China; ^2^Liangshan Zhou Company of Sichuan Province Company of Tobacco Corporation in China, Liangshan Zhou, China; ^3^Sichuan Province Company of Tobacco Corporation in China, Chengdu, China; ^4^Panzhihua City Company of Sichuan Province Company of Tobacco Corporation in China, Panzhihuan, China; ^5^Key Laboratory of the Development and Resource Utilization of Biological Pesticide in Inner Mongolia, Hohhot, China

**Keywords:** tobacco, tabacco vein molting virus, RNA-Seq, GFP, 3′UTR

## Abstract

**Introduction:**

Tobacco vein mottling virus (TVMV) is a member of the family *Potyviridae*. The 3’ untranslated region (3’UTR) of viral genomic RNA has been reported to significantly impact viral infection. Nevertheless, the role of the TVMV 3’UTR during viral infection remains unknown.

**Methods:**

Here, a 3’UTR-GFP expression vector was transiently expressed in *Nicotiana benthamiana*, in which the 3’UTR of TVMV was introduced upstream of the green fluorescent protein (GFP) gene. Transcriptome sequencing was performed to analyze the genes associated with plant resistance. The effect of the TVMV 3’UTR on GFP expression was studied using an *Agrobacterium*-mediated transient expression assay, revealing that the TVMV 3’UTR significantly inhibited GFP expression. Transcriptome analysis of differentially expressed genes in 3’UTR-GFP in *N. benthamiana* was performed to elucidate the why the TVMV 3’UTR inhibited GFP expression.

**Results:**

Eighty genes related to plant disease resistance were differentially expressed, including 29 upregulated and 51 downregulated genes. Significantly upregulated genes included those encoding the calcium-binding protein CML24, leucine-rich repeat receptor-like tyrosine-protein kinase, and respiratory burst oxidase homolog protein E. The significantly downregulated genes included calcium-binding protein 7, ethylene-responsive transcription factor 10, endoglucanase 5, and receptor-like protein kinase.

**Discussion:**

These findings indicate that the 3’UTR of TVMV may inhibit the expression of GFP gene by inducing the expression of plant resistance genes. This study provides a theoretical basis for further research on the function and mechanism of the TVMV 3’UTR.

## 1 Introduction

A multitude of gene products derived from viral genomes, including coat proteins, replicases, and non-coding regions, have the capacity to induce resistance in plant hosts against pathogens (Abel et al., 1986; Golemboski and Zaitlin, 1990; Kollàr et al., 1993). Double-stranded RNA that express or synthesize the complete genes of plant viruses *in vitro* have also been demonstrated to induce plant resistance (Francisco et al., 2003). To infect plants, the virus must replicate in the initially inoculated cells, move between cells, and transfer to other parts of the host plant. Alongside viral replication-and viral motility-related proteins, the 3′ untranslated region (3'UTR) of viral genomic RNA also plays an important role in viral infection (Dong et al., 2015; Shivaprasad et al., 2015).

Tobacco vein mottling virus (TVMV) is a positive-sense single-stranded RNA virus belonging to *Potyvirus* (Sun et al., 2010; Domnier et al., 1986; Pirone and Gooding, 1973). Its genome is similar to that of potato Y virus. TVMV contains approximately 9,700 nucleotides (nt) and poly (A) tails. The lengths of 5′UTR and 3'UTR are 152 nt and 253 nt, respectively. The 3'UTR of positive single-stranded RNA viruses is highly structured. Multiple elements in this region interact with other nucleotides or proteins from viruses and cells to regulate various aspects of the viral life cycle, such as replication, translation, and host cell responses. Research on virus pathogenesis and the interactions between the virus and host can provide a reference for the study of plant-positive single-stranded RNA viruses and potato virus disease.

A substantial body of research has demonstrated that the expression of specific viral sequences in genetically modified plants confers protection against subsequent viral infections. The 3'UTR sequence of cucumber mosaic virus (CMV) has a highly conserved nucleotide sequence and a very important secondary structure for CMV replication (Kwon and Chung, 2000; Rietveld et al., 1983). Duan and Guo (2008) used the 3'UTR of CMV to design hairpin RNA, which was transformed into tobacco to produce constitutive virus siRNA. The transgenic plants exhibited delayed resistance to CMV infection and recovery phenotypes. Furthermore, the introduction of the 3'UTR region of turnip yellow mosaic virus into the rapeseed genome showed a partially protective effect. Nevertheless, as the inoculation concentration increased, this effect was diminished (Haenni et al., 1993). This suggests that the observed protective effect was likely due to competition for viral replicase. A comparable mechanism of competition has been proposed to explain the inhibition of brome mosaic virus replication in protoplasts by different regions of the virus RNA-bearing replication origin (Marsh et al., 1991a; Marsh et al., 1991b; Huntley and Hall, 1993). In addition, transcripts can prevent viral infection by forming RNA–RNA hybrids with (−) strands (Haenni et al., 1993).

The virus-induced production of resistance genes in host plants represents a significant area of interest in the broader field of plant virus-induced resistance mechanisms. Although numerous regions of the viral genome have been demonstrated to elicit plant resistance, there is a paucity of studies exploring the functions of the non-coding regions. The precise mechanism by which the 3'UTR of the virus contributes to the antiviral response in plants remains unclear. In this study, we constructed a 3'UTR sequence derived from TVMV and inserted it upstream of green fluorescent protein (GFP), resulting in the generation of a pEAQ-3'UTR-GFP expression vector. This was accomplished using gateway recombination technology. The effect of 3'UTR of TVMV on GFP expression was studied by transient expression in *Nicotiana benthamiana*. The differential gene expression after transient 3'UTR-GFP expression of *N. benthamiana* was analyzed by transcriptome sequencing. The objective of this study is to examine the function of the 3'UTR of TVMV and its role in plant disease resistance. It offers a theoretical foundation for understanding the function of TVMV 3'UTR.

## 2 Materials and methods

### 2.1 Experimental materials

The vectors pDONR207, pEAQ-HT-DEST3, and *Agrobacterium tumefaciens* C58C1 were provided by Professor Juan Antonio García from the National Centre for Biotechnology (CNB), Spain. *Escherichia coli* DH5α competent cells were purchased from TaKaRa Bio (Osaka, Japan). The infection clone of TVMV was described by Zhao et al. (2020).

### 2.2 Plant preparation

Seeds of *N. benthamiana* plants were sown in soil, and the plants were cultured in a greenhouse at 22°C under a 16/8 h light/dark cycle. In general, 4 ~ 5-leaf-stage *N. benthamiana* plants with uniform growth and size were selected for the experiments.

### 2.3 Construction of TVMV 3'UTR-GFP

Specific primers were designed according to the sequences of the 3'UTR of TVMV, GFP, and the expression vector pEAQ-HT-D3. The primers used are listed in Supplementary Table 1. High-fidelity PrimeSTAR GXL DNA polymerase (TaKaRa) was used to amplify fragments in overlap PCR. The TVMV infection clone, pLXB-TVMV, was described by Zhao et al. (2020). To facilitate fragment recombination, an overlapping segment of 24 bp in length was designed at the 3' and 5' ends of each primer. Two primer pairs (attB-3'UTR-F1/3UTR-GFP-R1 and 3'UTR-GFP-F2/attB-GFP-R2) were used to amplify the PCR products of the TVMV 3'UTR and GFP using pLXB-TVMV and pEAQ-GFP (Sun et al., 2023) as templates. The PCR fragments of the TVMV 3'UTR and GFP were mixed at a ratio of 1:1 as the template, and primers attB-3'UTR-F1 and attB-GFP-R2 were used to amplify the3'UTR-GFP fragment using TaKaRa LA Taq DNA polymerase. The three PCR products were detected and purified using 1% agarose gel electrophoresis.

The expression vector pEAQ-3'UTR-GFP was constructed by Gateway technology. The obtained PCR product of 3'UTR-GFP was subjected to BP reaction to construct an entry clone. Then, 3.5 μl purified PCR product, 0.5 μl donor vector pDONR207, and 1 μl BP enzyme mix were fully mixed and incubated at 25°C for 6 h. Subsequently, 5 μl of all BP reaction products was transformed into *E. coli* DH5α. The positive antibody was screened on LB solid medium containing 50 μg/ml gentamicin. Plasmid pDONR-3'UTR-GFP was extracted and detected with restriction endonucleases *Eag I* and *Pst I*. The plasmid was purified and sequenced using BGI Group (Shenzhen, China).

The obtained pDONR-3'UTR-GFP and the target vector PEAQ-HT-DEST3 were used for LR reaction. Then, 0.35 μl pDONR-3'UTR-GFP (100 ng), 1 μl target vector pEAQ-HT-DEST3 (100 ng), 1 μl LR enzyme mix, and 2.65 μl ddH_2_O were fully mixed and incubated at 25°C for 6 h. Next, 5 μl of all LR reaction products was transformed into *E. coli* DH5α. The positive antibody was screened on LB solid medium containing 50 μg/ml kanamycin. The plasmid PEAQ-3'UTR-GFP was extracted, purified, and sequenced by BGI Group.

### 2.4 Transient expression of 3'UTR-GFP in *N. benthamiana*

The constructed expression vector, pEAQ-3'UTR-GFP, was transformed into *Agrobacterium* C58C1 and expressed transiently in *N. benthamiana*. GFP expression was detected in the leaves. The cultured *Agrobacterium* C58C1 cells containing pEAQ-3'UTR-GFP were suspended in induction buffer (0.01 M MES solution, 0.01 M MgCl_2_, 0.15 mM Aceto mixed solution). The induced pEAQ-3'UTR-GFP bacterial suspension was injected into the third and fourth leaves of *N. benthamiana* from the back of the leaves with a 1 ml single-use aseptic injector to fully infiltrate the leaves. Then, the inoculated *N. benthamiana* was cultured at 22°C. *N. benthamiana* injected with pEAQ-GFP and pEAQ-HT-DEST3 were used as controls, with six plants per treatment. The expression of GFP in the leaves was observed 48 h after infection. The inoculated leaves were collected and flash frozen in liquid nitrogen to detect GFP expression.

### 2.5 Western blotting for GFP accumulation

Total protein was extracted using liquid nitrogen urea lysate via high-speed centrifugation. The plant samples were ground in a precooled mortar and pestle and collected in 1.5 ml centrifuge tubes. Protein lysate (pH = 7.5; 125 mM TrisHCl, 20% sodium dodecyl sulfate (SDS), 6 M urea, 5% β-mercaptoethanol, and a small amount of bromophenol blue) was added to the centrifuge tube at a 2:1 ratio, and the mixture was incubated in a 95°C metal bath for 10 min, followed by 2 min on ice. The centrifuge tubes were then centrifuged at 13,400 × *g* for 10 min at 4°C. Five microliters of the prepared protein samples were subjected to sodium dodecyl sulfate–polyacrylamide gel electrophoresis. Following electrophoresis, the membrane transfer process was initiated, and the protein was transferred to a nitrocellulose membrane and blocked in a blocking solution for 2 h. The nitrocellulose membrane was washed with TBST and incubated with 5,000-fold-diluted GFP monoclonal antibody (Bio-Rad, Hercules, California, USA), before being washed again with TBST and incubated with 5,000-fold-diluted alkaline phosphatase-labeled goat anti-rabbit IgG (Bio-Rad, Hercules, California, USA) secondary antibody. Then, the membrane was washed a third time with TBST and treated with an ECL color development solution (Bio-Rad, Hercules, California, USA) to visualize the protein bands on an Odyssey® Fc near-infrared dual-color laser and chemiluminescence dual-imaging function imaging system.

### 2.6 RT-qPCR analysis for GFP expression

TRIzol reagent was used to extract total RNA and further synthesize cDNA (TransGen Biotech, Beijing, China). Based on the sequence of the GFP coding region, Primar5.0 was used to design qPCR primers. The upstream primer was GFP (el)-F, and the downstream primer was GFP (el)-R. GFP expression was detected by quantitative real-time PCR (RT-qPCR). *NtUB1* was used as the internal reference gene, and its upstream and downstream primers were NtUB1-F and NtUB1-R, respectively (Supplementary Table 1). Three replicates were analyzed for each gene. The 2^−ΔΔCT^ method was used to calculate the level of gene expression.

### 2.7 RNA library construction and sequencing of *N. benthamiana* after 3'UTR-GFP expression

Total RNA was extracted using TRIzol reagent (Thermo Fisher Scientific, Waltham, Massachusetts, USA) following the manufacturer’s instructions. Total RNA quantity and purity were determined using a Bioanalyzer 2100 and RNA 6000 Nano LabChip Kit (Agilent Technologies, Santa Clara, California, USA), and high-quality RNA samples with RNA integrity number > 7.0 were used to construct the sequencing library. RNA libraries were sequenced on an Illumina Novaseq 6000 platform in PE150 mode by LC Bio Technology Co., Ltd. (Hangzhou, China). The transcriptome comprised nine cDNA libraries, consisting of three biological replicates and three sets of treatments.

Raw data generated by sequencing were preprocessed and filtered to obtain clean data. Clean data were compared with the *N. benthamiana* genome^1^ to obtain comprehensive alignment information. Simultaneously, according to the gene location information specified in the genome annotation file gtf, the following statistics were performed: read comparison between the sequencing data and the reference genome and the regional distribution analysis of the sequencing data and the reference genome. The quantitative status of each sample gene was determined using StringTie. After quantitative gene analysis, differentially expressed genes (DEGs) were screened for functional annotation and enrichment analysis. Gene expression was expressed as fragments per kilobase million. Fold change ≥2 and false discovery rate < 0.01 were used as screening criteria. The raw sequence data were submitted to the NCBI Short Read Archive (SRA) with accession number PRJNA1134345.

### 2.8 DEG analysis of *N. benthamiana* after 3'UTR-GFP expression

EdgeR was used to analyze the DEGs in *N. benthamiana* after the transient expression of 3'UTR-GFP, GFP, and Vector for 48 h. The number of up-and downregulated genes was obtained by using |log_2_FC| ≥ 1 and *q* < 0.05 as the screening criteria. Comparison and annotation were performed using the Gene Ontology (GO) database, and GO functional annotations were obtained using ‘Blast2GO’ software (Conesa et al., 2005). All GO functional annotations were classified using ‘WEGO’ software (Ye, 2006), and the DEGs were assigned to cellular component, biological process, and molecular function categories in the GO database. Pathway enrichment analysis was performed using the Kyoto Encyclopedia of Genes and Genomes (KEGG) database, the main public pathway database, which primarily analyzes the metabolic pathways and functions of cellular proteins. The KEGG database was used to annotate the metabolic pathways of each independent gene translational protein.

### 2.9 RT-qPCR validation of DEGs

Nine DEGs of *N. benthamiana* after transient 3'UTR-GFP expression were selected for RT-qPCR verification, and the necessary primers were synthesized by Shanghai Sangon Biological Co. (Supplementary Table 1). The *NtUB1* gene was used as an internal reference gene, and cDNA templates were obtained from the transcriptome sequencing samples. The 2^−ΔΔCT^ method was used to determine the relative expression.

## 3 Results

### 3.1 Construction of 3'UTR-GFP

**Figure 1:**
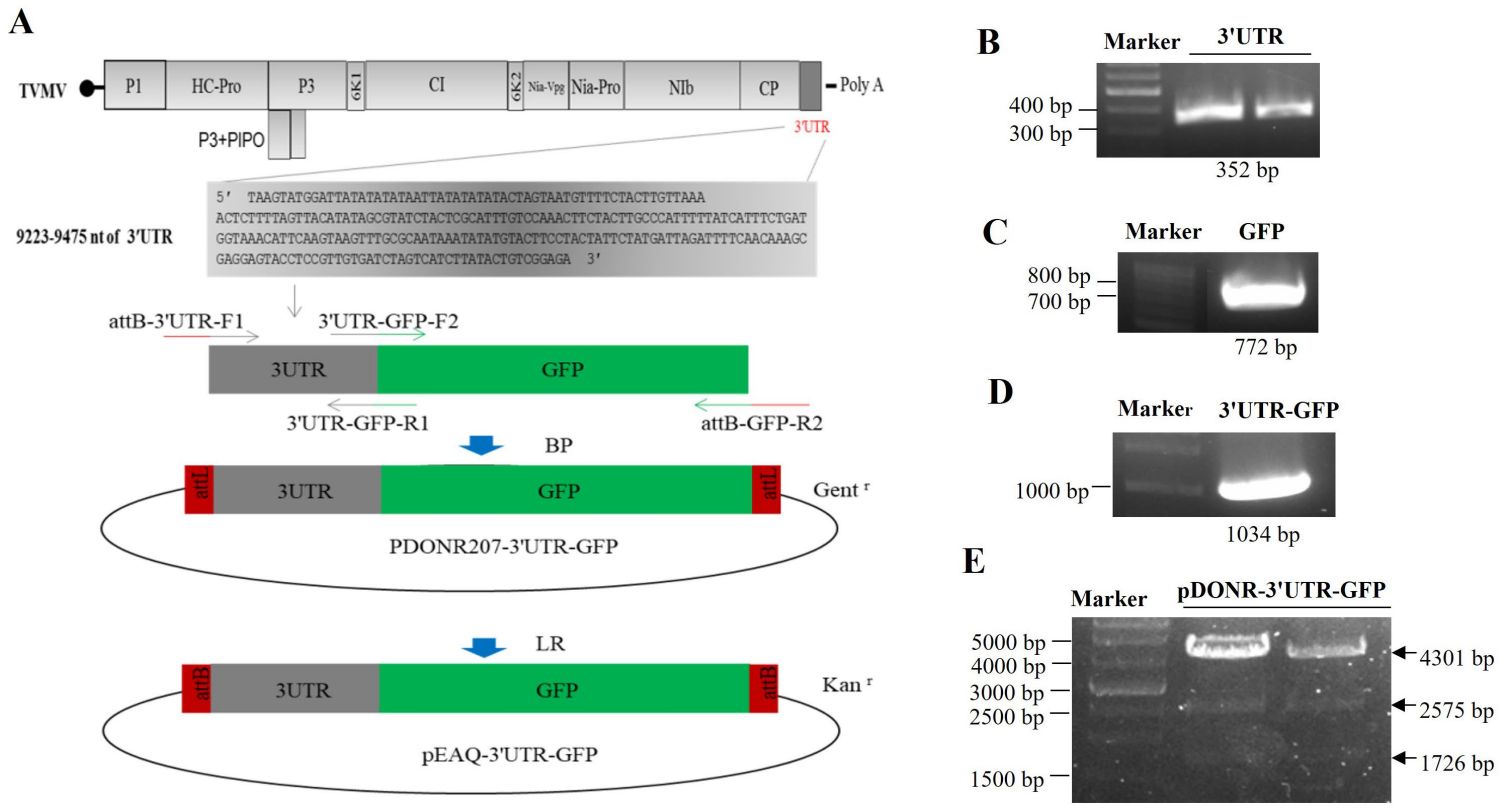
Construction of the 3'UTR-GFP. **(A)** Schematic of 3'UTR-GFP construction; **(B)** 3'UTR fragments (352 bp) obtained by PCR amplification; **(C)** GFP fragments (772 bp) obtained by PCR amplification; **(D)** 3'UTR-GFP gene fragments obtained by PCR amplification; **(E)** digestion verification of pDONR-3'UTR-GFP by *Eag I* and *Pst I*.

**Figure 2:**
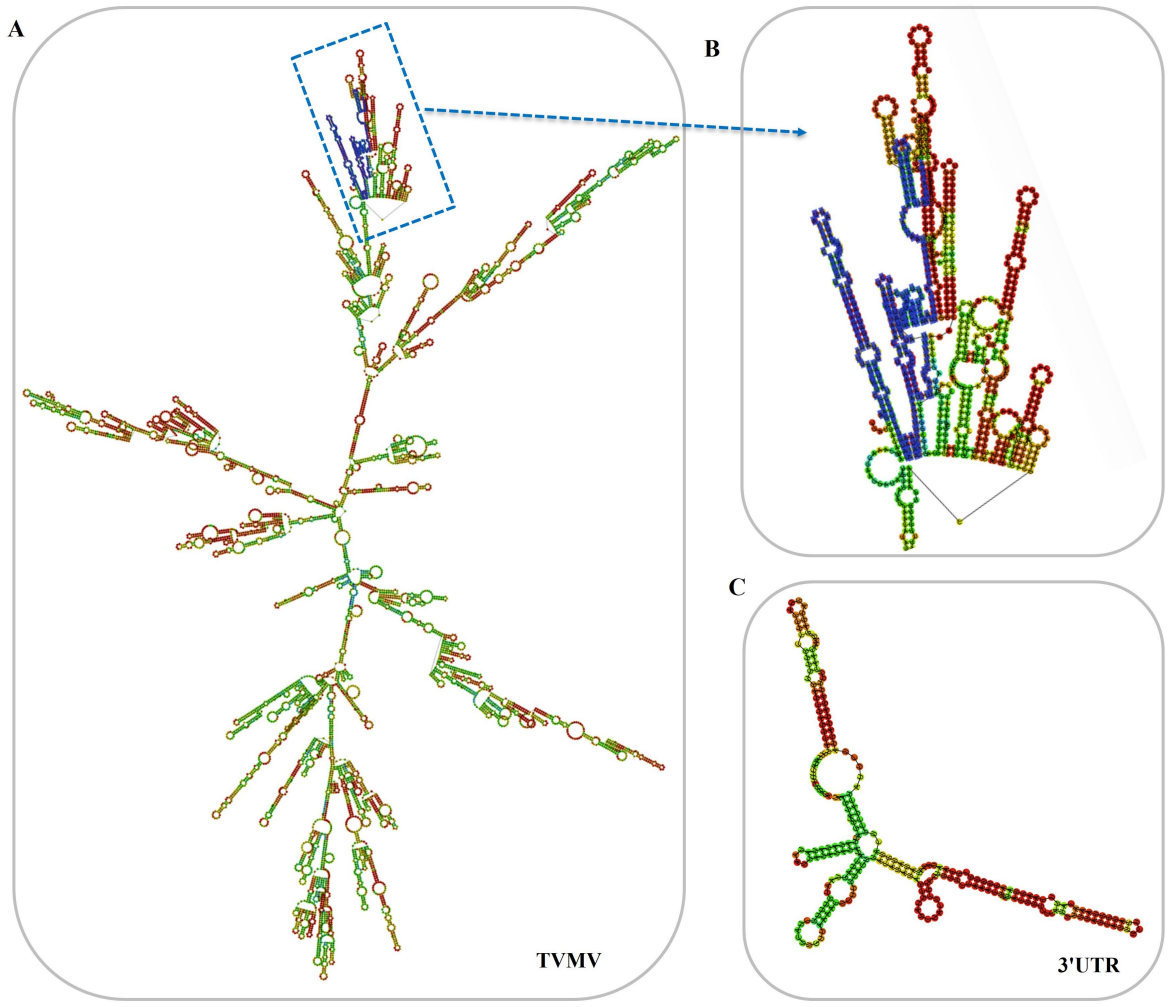
Predicted secondary structures of 3'UTR *in vitro*. **(A)** Predicted secondary structures of TVMV(7,500 nt from 3'end); **(B)** Predicted secondary structures of 3'UTR in the virus, the 3'UTR sequences were labeled in blue; **(C)** Predicted secondary structures of 3'UTR *in vitro* (253 nt).

The 3'UTR-GFP construction was conducted in accordance with the Gateway system (Figure 1A). To obtain the 3'UTR-GFP fragment, fragments of 3'UTR (352 bp) and GFP (772 bp) of TVMV were amplified (Figures 1B,C). Then, the 3'UTR of TVMV was fused upstream of GFP by overlap PCR to create the 3'UTR-GFP (1,034 bp; Figure 1D). Next, 3'UTR-GFP was introduced into the expression vector pEAQ-HT-D3 via the Gateway recombination system. First, the 3'UTR-GFP fragment was cloned into the entry clone pDONR207 by BP reaction. pDONR-3'UTR-GFP was digested by *Eag I* and *Pst I*, which resulted in three bands of 4,301, 2,575, and 1,726 bp in length (Figure 1E). Then, the 3'UTR-GFP fragment was introduced into the expression vector pEAQ-HT-D3 via LR reaction, resulting in the creation of pEAQ-3'UTR-GFP. The plasmids pDONR-3'UTR-GFP and pEAQ-3'UTR-GFP were subjected to sequencing, and the 3'UTR-GFP sequences in each plasmid were subsequently analyzed (Supplementary Figure 1; Supplementary Table 2). The 3'UTR-GFP sequence was found to be accurate and uniform across all plasmids, as evidenced by the sequencing peak figures (Supplementary Figure 2; Supplementary Table 2). In order to check if the 3'UTR structure in pEAQ vector is the same as that of TVMV virus. We have predicted the structure of 3'UTR *in vitro* by RNAfold Webserver and make a comparison with the structure in TVMV viral RNA. We have used the 253 nt of 3'UTR to perform the structure prediction. Regarding with the TVMV virus, the limited length of RNA for structure prediction is 7,500 nt. We choose the 7,500 nt of TVMV calculated from 3' end. It appears that the structure of 3'UTR alone and that of in virus are similar (Figure 2). It is supposed that the 253 nt of 3'UTR will be transcribed in pEAQ-D3-3'UTR after agro-infiltrated in *N. benthamiana*.

### 3.2 Influence of transient 3'UTR-GFP expression on GFP fluorescence in *N. benthamiana*

**Figure 3:**
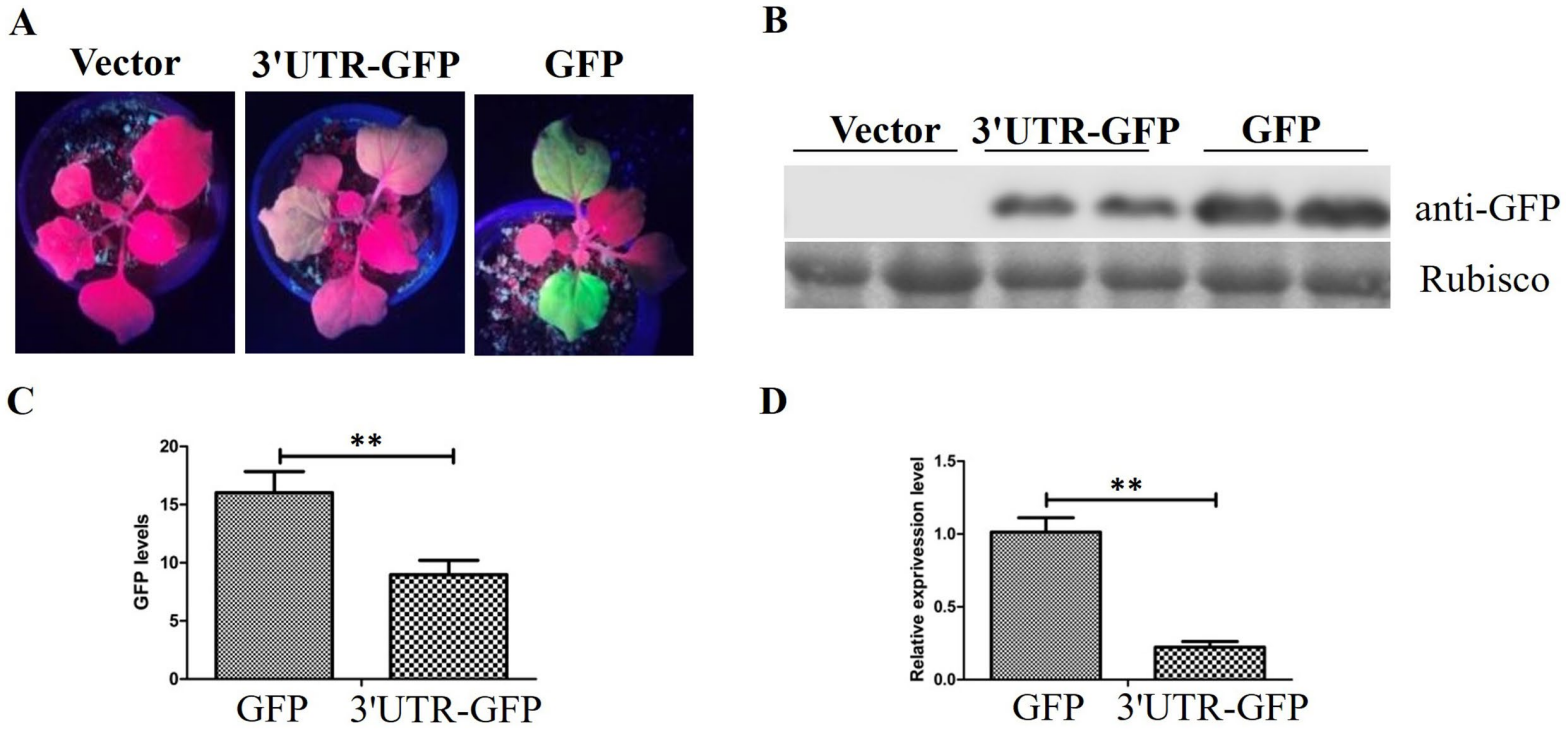
GFP expression in *N. benthamiana*. **(A)** GFP fluorescence in *N. benthamiana* detected 48 h after injection of pEAQ-3'UTR-GFP (3'UTR-GFP) and pEAQ-GFP (GFP); **(B)** Western blot analysis of GFP expression by anti-GFP using the Rubisco protein band as loading control; **(C)** quantitative analysis of GFP signal in the Western blot assay; **(D)** relative expression of GFP after transient expression of 3'UTR-GFP and GFP, detected by RT-qPCR. ** represents *p* < 0.01 by Student’s *t* test.

The pEAQ-3'UTR-GFP expression vector was transformed into *Agrobacterium* C58C1 and inoculated into leaves of *N. benthamiana*. GFP expression was observed 48 h after injection. Pronounced green fluorescence was evident on *N. benthamiana* leaves that were injected with pEAQ-GFP, whereas the fluorescence on leaves injected with pEAQ-3'UTR-GFP was considerably weaker (Figure 3A).

The inoculated leaves of *N. benthamiana* were harvested, and the protein extracts were utilized directly for Western blotting with anti-GFP antibody. The results demonstrated the absence of GFP protein in the injected leaf samples inoculated with the empty vector pEAQ-HT-D3. The level of GFP expression in samples treated with pEAQ-3'UTR-GFP was lower than that in samples receiving pEAQ-GFP (Figure 3B). The GFP signal was quantitatively analyzed, and the results are shown in Figure 3C. The GFP signal in the pEAQ-3'UTR-GFP samples exhibited a markedly diminished intensity in comparison to that observed in the pEAQ-GFP samples (*p* = 0.004; Figure 3C).

RT-qPCR was performed to confirm the difference in GFP expression between the two samples. GFP expression was in accordance with the results of Western blotting, as shown in Figure 3D. The GFP expression in pEAQ-3'UTR-GFP samples was significantly lower than that in pEAQ-GFP samples (*p* = 0.006).

### 3.3 Transcriptome analysis of DEGs in *N. benthamiana* infiltrated with 3'UTR-GFP

Transcriptome sequencing analysis of *N. benthamiana* leaves infiltrated with empty vector pEAQ-HT-D3, pEAQ-3'UTR-GFP, and pEAQ-GFP was performed using an Illumina Novaseq™ 6000 platform (LC Bio Technology CO., Ltd. Hangzhou, China). The three treatments yielded 5.17, 5.36, and 6.19 G clean reads, respectively. The G + C content of the bases was >42.5%, and the proportion of Q30 bases was >98.20% (Supplementary Table 3). The genome sequence of *N. benthamiana* was used as a reference. The clean reads of all the samples showed 89.63–92.39% similarity with the reference genome, as shown in Supplementary Table 3. These findings confirm the accuracy and reliability of the sequencing assembly results, making them suitable for further analyses.

The distribution of gene expression values across the samples revealed that the nine boxes were similar in size, indicating a high degree of similarity in gene density among the nine samples. The consistency in whisker lengths demonstrated that samples exhibited similar variance. The median line of the samples was closer to the high values of the boxes, with a long whisker shape extending towards the low values, thereby producing a negative bias (Supplementary Figure 3A). A correlation analysis of the gene expression information from the 3'UTR-GFP, GFP, and Vector samples revealed that the Pearson correlation coefficient for all three groups of samples was above 0.9 (Supplementary Figure 3B), indicating a high degree of correlation in the expression patterns between the three groups. This confirms the reliability of the data, which can be used for further analyses.

### 3.4 Differential gene expression in *N. benthamiana* transiently expressing 3'UTR-GFP

**Figure 4:**
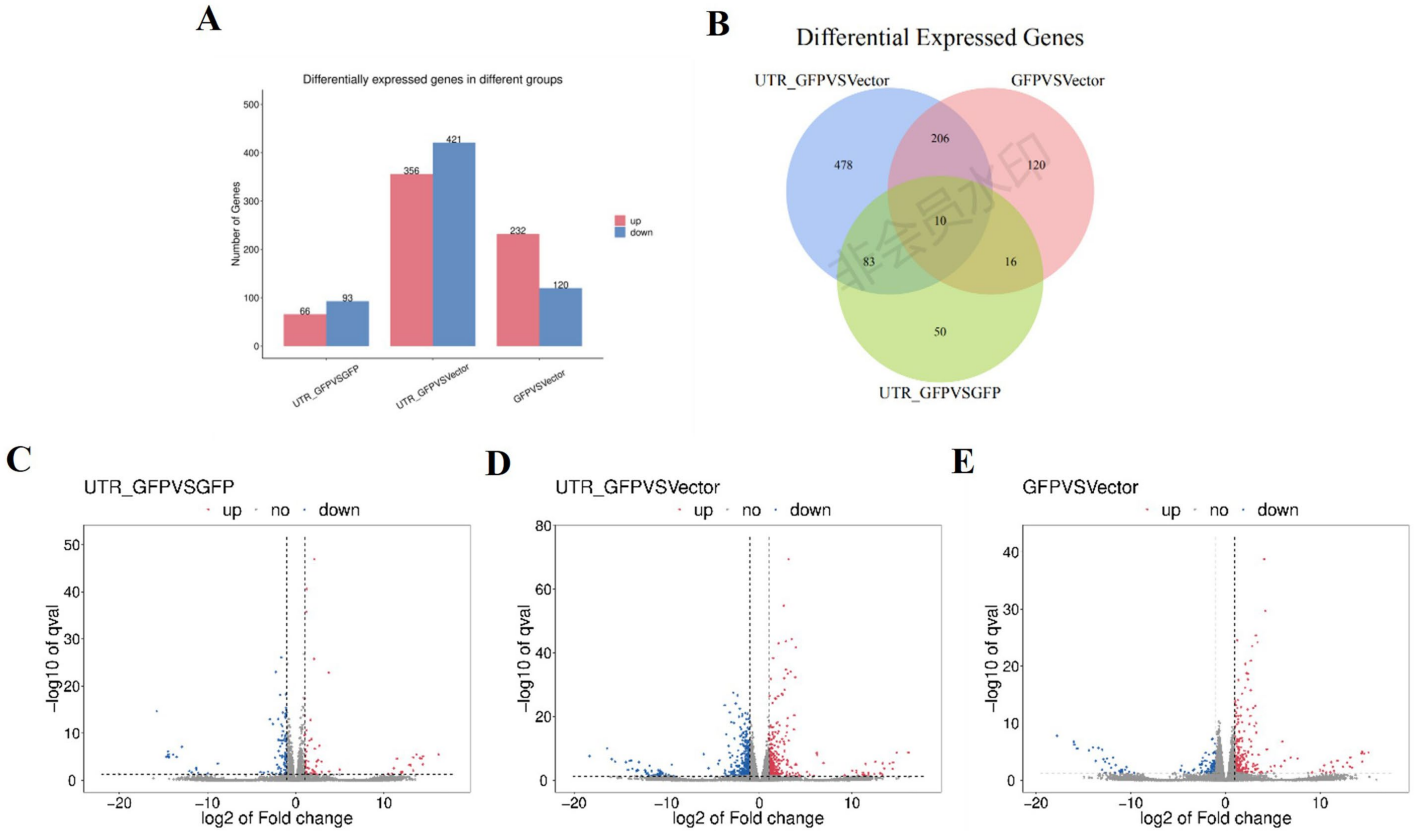
DEGs of 3'UTR-GFP transiently expressed in *N. benthamiana*. **(A)** Statistics of up-and downregulated DEGs; **(B)** volcano plot of 3'UTR-GFP vs. GFP DEGs. The abscissa represents the log_2_(FC) of the differential genes; the ordinate represents the *p*-value (−log_10_ q-value). Gray represents genes that are not differentially expressed compared with the control; red represents upregulated genes; and green represents downregulated genes. **(C)** Volcano plot of 3'UTR-GFP vs. Vector DEGs; **(D)** volcano plot of GFP vs. Vector DEGs; **(E)** Venn diagram of 3'UTR-GFPvsGFP, 3'UTR-GFPvsVector, and GFPvsVector DEGs. Each circle in the graph represents a comparison group, and the overlapping parts of the circle represent the DEGs shared between the comparison groups. The non-overlapping parts represent the number of DEGs specific to this group.

DESeq2 (Anders and Huber, 2010) was used to analyze the DEGs among 3'UTR-GFP vs. GFP, 3'UTR-GFP vs. Vector, and GFP vs. Vector samples, revealing 159, 777, and 352 DEGs, respectively (|log_2_FC| ≥ 1 and *q* < 0.05; Figure 4A; Supplementary Table 4). Overall, 66, 356, and 232 DEGs were upregulated and 93, 421, and 120 were downregulated in the 3'UTR-GFP vs. GFP, 3'UTR-GFP vs. Vector, and GFP vs. Vector comparisons, respectively (Figures 4C–E). Cluster analysis identified 10 DEGs common to all comparisons and 50, 478, and 120 unique DEGs in the 3'UTR-GFP vs. GFP, 3'UTR-GFP vs. Vector, and GFP vs. Vector comparisons (Figure 4B; Supplementary Table 5).

**Figure 5:**
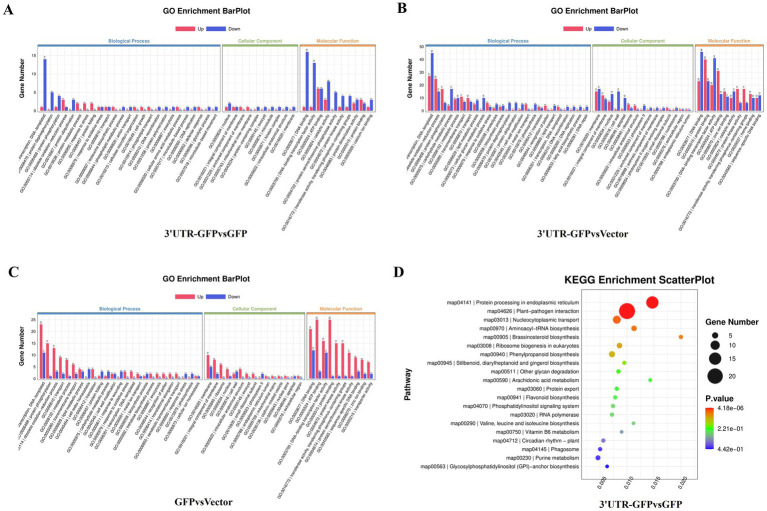
GO and KEGG analyses of DEGs in *N. benthamiana* after transient 3'UTR-GFP expression. **(A)** GO enrichment analysis of 3'UTR-GFP vs. GFP DEGs. The abscissa represents the GO functional annotation, the ordinate represents the number of DEGs involved in this function, and the number of upregulated and downregulated genes is shown in red and blue, respectively. **(B)** GO enrichment analysis of 3'UTR-GFP vs. Vector DEGs; **(C)** GO enrichment analysis of GFP vs. Vector DEGs; **(D)** KEGG enrichment analysis DEGs among 3′UT-GFP vs. GFP samples. The horizontal enrichment factor represents the ratio of DEGs in this pathway to the total number of genes enriched in this pathway. The greater the enrichment factor, the higher the enrichment degree of DEGs in this pathway. The ordinate is the KEGG pathway. The size of the bubble represents the number of DEGs in the corresponding pathway; the larger the bubble, the more DEGs there are in the pathway. The color of bubbles represents the *p*-value from enrichment analysis. The color ranges from blue to red. The redder the color, the smaller the *p*-value and the more significant the enrichment.

The 159 DEGs in the 3'UTR-GFP vs. GFP comparison were subjected to GO functional enrichment analysis and quantitative statistics (Figure 5A). We found 25 categories of biological processes, all of which showed differential gene enrichment. Among them, “regulation of transcription, DNA-templated” genes were significantly enriched, with one gene was upregulated in 3'UTR-GFP group relative to its expression in GFP group. Fourteen illogical process genes were downregulated in 3'UTR-GFP group relative to its expression in GFP group, accounting for 9.4% of the total downregulated genes. We identified 22 categories of cellular components, 10 of which showed differential gene enrichment. The “nucleus” and the “integral component of membrane “had the most gene enrichment, with one upregulated gene each and two and one downregulated genes, respectively. Differential gene enrichment was also observed for all 22 molecular function classifications. In particular, genes in “DNA binding,” “DNA-binding transcription factor activity,” “ATP binding,” and “catalytic activity” had the most gene enrichment, with one, one, six, and three upregulated genes, respectively. These genes accounted for 1.3, 1.3, 8, and 4% of the total upregulated genes, respectively. The number of downregulated genes was 16, 13, 6, and 8, accounting for 10.7, 8.7, 4.0, and 5.4% of the total downregulated genes, respectively.

The 777 DEGs in the 3'UTR-GFP vs. Vector comparison were also subjected to GO function enrichment analysis and quantitative statistics (Figure 5B). We found 25 biological process categories showing differential gene enrichment. Among them, “regulation of transcription, DNA-templated” genes had the most gene enrichment, with 27 upregulated and 45 downregulated genes in the 3'UTR-GFP group, accounting for 4.4 and 6.3% of the total up-and downregulated genes, respectively. All 22 cell components categories showed differential gene enrichment. Membrane gene enrichment was significant; the number of upregulated and downregulated genes was 15 and 17, respectively. Differential gene enrichment was also observed for all 22 molecular function classifications, with significant enrichment in genes related to “DNA binding,” “protein binding,” “DNA-binding transcription factor activity,” and “ATP binding,” with 23, 40, 20, and 31 upregulated genes accounting for 3.8, 6.5, 3.3, and 5.1% of the total upregulated genes, respectively. The number of downregulated genes was 46, 23, 41, and 13, representing 6.4, 3.2, 5.7, and 1.8% of the total downregulated genes, respectively.

Finally, GO functional enrichment analysis and quantitative statistics were performed for the 352 DEGs in the GFP vs. Vector comparison (Figure 5C). Transcriptional regulation and DNA template genes had the most gene enrichment, with 23 upregulated genes accounting for 5.0% of the total upregulated genes. Eleven genes were downregulated, representing 6.6% of all the downregulated genes. Among the 22 cell component categories, 16 were enriched in DEGs. Membrane genes had the most gene enrichment, with 10 upregulated and 15 downregulated genes. Differential gene enrichment was observed for all 22 molecular function classifications. Genes related to “DNA binding,” “ATP binding,” “DNA-binding transcription factor activity,” and “protein binding” had the most gene enrichment, with 10, 8, 6, and 4 upregulated genes accounting for 2.2, 1.8, 1.3, and 0.9% of the total upregulated genes, respectively. In contrast, five, two, zero, and one genes were downregulated, respectively.

KEGG pathway analysis of the DEGs in the 3'UTR-GFP group vs. the GFP group annotated 98 DEGs (61% of all genes), of which 91 had the most gene enrichment. The enrichment level of DEGs in the plant–pathogen interaction pathway was the highest, with 18 DEGs enriched, 3 of which were upregulated and 15 downregulated (Figure 5D; Supplementary Table 6).

**Figure 6:**
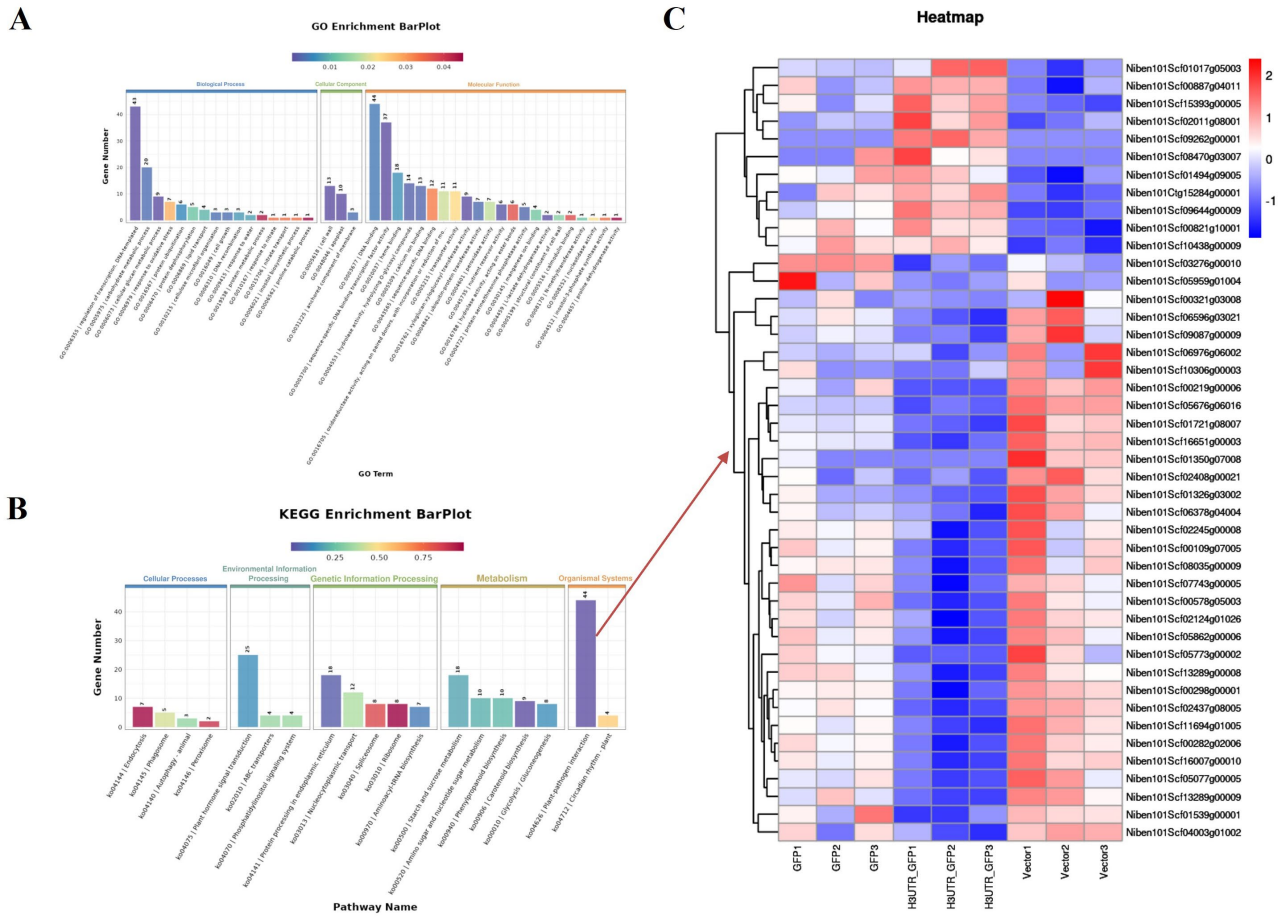
Enrichment analysis of DEGs related to 3'UTR reaction in *N. benthamiana*. **(A)** GO functional annotation classification statistical map; **(B)** KEGG enrichment analysis statistical map; **(C)** heat map of the transcript levels of DEGs enriched in the plant–pathogen interaction pathway. Relative expression is shown as a color gradient from low (blue) to high (red).

By removing the DEGs in the GFP vs. Vector comparison from those obtained in the 3'UTR-GFP vs. GFP and 3'UTR-GFP vs. Vector comparisons, we identified 611 DEGs related to 3'UTR response (Supplementary Table 7). These 611 DEGs were subjected to GO functional enrichment analysis, with results presented in Figure 6A. Overall, 350 genes were significantly enriched in 41 GO terms (*p* < 0.05). In the biological process branch, the two terms containing the most DEGs were “transcriptional regulation, DNA-templated” and “carbohydrate metabolic processes.” In the cellular component branch, the three terms containing DEGs were “cell wall,” “apoplast,” and “anchored component of the membrane.” In the molecular function branch, the terms containing the most DEGs were “DNA binding,” “sequence-specific DNA-binding transcription factor activity,” and “heme-binding.”

The results of KEGG signaling pathway enrichment showed that 269 DEGs were enriched in 95 KEGG pathways, of which 6 were significantly enriched (*p* ≤ 0.05). As shown in Figure 6B, the plant–pathogen interaction (ko04626) pathway had the highest enrichment level of DEGs, with 44 DEGs annotated to the pathway, of which 11 were upregulated and 33 were downregulated in the 3'UTR-GFP group (Figure 6C; Supplementary Table 8).

**Figure 7:**
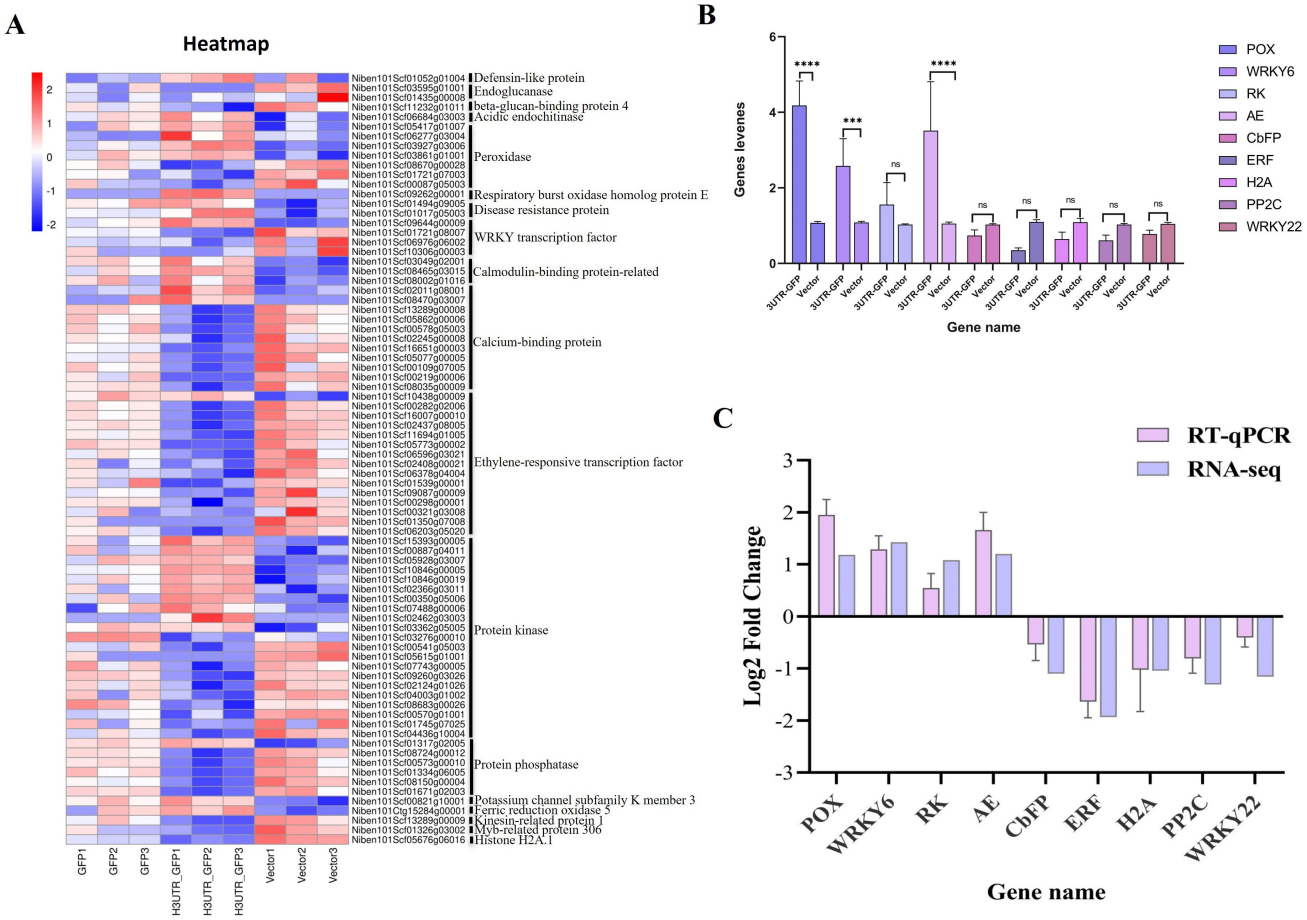
Heat map of the DEGs related to plant resistance and RT-qPCR validation. **(A)** Heat map of the transcript levels of the 80 DEG related to plant resistance. Relative expression is shown as a color gradient from low (blue) to high (red). **(B)** Relative expression of DEGs after the transient expression of 3'UTR-GFP and GFP, as analyzed by RT-qPCR. ****p < 0.0001, ***p < 0.0007, and “ns” represents *p* > 0.1 by Student’s *t* test. **(C)** Verification of DEGs by RT-qPCR.

Among the 611 DEGs related to the 3'UTR reaction, we obtained 80 related to plant resistance, of which 29 were upregulated and 51 were downregulated (Figure 7A; Supplementary Table 9). The upregulated genes included those encoding defensin-like protein 3, acidic endochitinase, peroxidase, respiratory burst oxidase homolog protein E, disease resistance protein, WRKY transcription factor 6, calcium-binding protein, calmodulin-binding protein, ethylene-responsive transcription factor 4, protein kinase, protein phosphatase 2C 35, potassium channel subfamily K member 3, and ferric reduction oxidase 5. The downregulated genes included those encoding endoglucanase, beta-glucan-binding protein 4, peroxidase, WRKY transcription factor, calcium-binding family protein, ethylene-responsive transcription factor, protein kinase, protein phosphatase 2C family protein, kinesin-related protein 1, Myb-related protein 306, and histone H2A.1.

### 3.5 Candidate genes and RT-qPCR verification

Nine DEGs, namely, peroxidase 5 (*POX*), WRKY transcription factor 6 (*WRKY6*), receptor kinase 2 (*RK*), acidic endochitinase (*AE*), calcium-binding EF-hand family protein (*CbFP*), ethylene-responsive transcription factor 10 (*ERF*), histone H2A.1 (*H2A*), protein phosphatase 2C family protein (*PP2C*), and WRKY transcription factor 22 (*WRKY22*), were selected for RT-qPCR verification. Each gene signal was quantitatively analyzed, and the results are shown in Figure 7B. Compared with their expression in the Vector group, four genes were upregulated and five were downregulated in the 3'UTR-GFP group, of which three upregulated genes were significantly differentially regulated. The Log_2_FC value was calculated using the 2^−ΔΔCT^ method. The results showed that the expression patterns of the nine selected genes were consistent with the transcriptome data compared to the RNA-seq data (Figure 7C). This finding corroborated the reliability of the transcriptome data.

## 4 Discussion

Plant viral diseases are extremely harmful to crops and can cause problems such as crop yield reduction, substantial economic losses, and grave threats to agricultural production. The 3'UTR of plant viruses plays an important role in the process of viral infection. The non-coding regions of some viruses have been reported to induce pathogen-induced resistance in plants (Xue et al., 2002). Here, the 3'UTR of TVMV was used as the experimental object and was constructed upstream of GFP to test if the 3'UTR could affect the GFP expression. The transcriptome of *N. benthamiana* after transient 3'UTR-GFP expression was analyzed using RNA-seq technology, DEGs were identified, and functional annotation and classification were performed. The results showed that the TVMV 3'UTR may affect the expression of GFP by regulating the expression of plant resistance-related genes.

By subjecting the obtained data to screening and identification procedures, we obtained information regarding genes that are differentially expressed in response to 3'UTR stimuli. KEGG enrichment analysis revealed that the DEGs in the plant–pathogen interaction pathway exhibited a markedly elevated level of enrichment, representing the most significantly enriched pathway. The WRKY transcription factor (WRKY TF) 6 gene had the highest significance (FDR = 2.32 × 10^−17^) and was upregulated by log_2_ (FC) = 1.42, whereas WRKY TF 22, 18, and 1 genes were downregulated. WRKY TFs are among the largest families of transcriptional regulators in plants and play critical roles in plant processes in response to biotic and abiotic stresses (Jiang et al., 2016). In terms of plant defense, WRKY TFs play important roles as positive and negative regulators via transcriptional regulation or protein–protein interactions (Huh et al., 2012). For example, *Arabidopsis* WRKY DNA-binding protein 30 (WRKY30), which was induced by CMV infection, played a positive regulatory role in plant CMV resistance. *WRKY30* mutants displayed greater disease susceptibility, while *WRKY30*-overexpressing plants exhibited greater resistance to CMV infection (Zou et al., 2019). Furthermore, *OsWRKY7* in rice functions as a crucial positive regulator of basal immunity against *Xanthomonas oryzae* pv. *oryzae* (*Xoo*; Zheng et al., 2024). WRKY TFs in cassava play a role in regulating plant tolerance and susceptibility to cassava mosaic disease through their involvement in stress responses (Freeborough et al., 2021).

In the plant–pathogen interaction pathway, the ethylene-responsive transcription factor (*PpERF*) 10 gene of *N. benthamiana* expression of 3'UTR-GFP was downregulated by log_2_ (FC) = −1.46. In addition, 13 *PpERF* genes were downregulated, and 1 was upregulated. The Apetala2/ethylene-responsive factor (*AP2/ERF*) transcription factors represent a large group of factors that are mainly found in plants (Feng et al., 2020). These transcription factors serve as important regulators of many biological and physiological processes and play notable roles in regulating gene expression under abiotic and biotic stresses in the plant kingdom (Gao et al., 2014; Yang et al., 2021). For instance, cotton ethylene response factor 6 (*GhERF6*), with the major defense-related latex protein GhMLP28 from upland cotton, facilitates the binding of *GhERF6* to the GCC-box element. *GhMLP28* acts as a positive regulator of *GhERF6*, and the synergetic actions of the two proteins may contribute substantially to the protection against *Verticillium dahliae* infection in cotton plants (Yang et al., 2015).

Several genes in the present study were associated with calcium-binding protein-related genes, with four upregulated and nine downregulated genes, the most notable of which was *CbFP* by log_2_ (FC) = −1.38. Calcium is an important part of plant immune signaling essential for activating host resistance (Liu et al., 2022). Calcium ions (Ca^2+^) are prominent intracellular messengers in all eukaryotic cells and play a crucial role in plant immunity. Ca^2+^ signaling is an early and necessary event in plant immunity (De et al., 2013). Calmodulin-binding TFs are involved in the regulation of plant responses to biotic stress (Zeng et al., 2023). Tobacco mosaic virus (TMV) coat protein-interacting protein L is associated with calmodulin-like protein 30 (NbCML30) in the cytoplasm and nucleus; NbCML30 silencing promotes TMV infection, whereas its overexpression inhibits TMV infection by activating Ca^2+^-dependent oxidative stress in plants (Liu et al., 2022). Ca^2+^ signaling to related to RNAi and wounding in *N. benthamiana* cells during viral infection activates RNAi-related gene expression, and calmodulin knockdown/knockout plants show increased susceptibility to geminiviruses, cucumoviruses, and *Potyvirus* (Wang et al., 2021).

The significance of DEGs related to plant resistance was analyzed, and the *PP2C* gene had the most significant differential expression (FDR = 1.0311 × 10^−23^) and was downregulated by log_2_ (FC) = −2.25. In addition, four *PP2C* family genes were downregulated, and one was upregulated. *PP2Cs* have been demonstrated to play critical roles in the regulation of plant growth and development (Kamenetsky et al., 2015), the abscisic acid signaling pathway (Rodriguez, 1998), and stress signaling (Fuchs et al., 2013). Some reports have concluded that the abscisic acid-induced *PP2C* signaling pathway is related to defense (Lu et al., 2019). For instance, *OsBIPP2C2a* overexpression in transgenic tobacco plants may play an important role in increasing disease resistance against TMV and *Phytophthora parasitica* var. *nicotianae* through the activation of the defense response (Hu et al., 2009).

The receptor-like protein kinase gene was highly significantly (FDR = 4.16 × 10^−15^) upregulated by log_2_ (FC) = 1.00. Several DEGs were associated with protein kinase-related genes, with 10 upregulated and 11 downregulated genes. Receptor kinases are proteins localized on the cell surface. In plants, these proteins are known as receptor-like kinases and play a prominent role in plant–pathogen interactions (Zhou et al., 2017). Several of these kinases influence plant susceptibility to viruses and, in some cases, interact with viral proteins (Macho and Lozano-Duran, 2019). The receptor-like kinase BAM1 from *Arabidopsis* interacts with TMV movement proteins to support the early movement of the virus and is required for the efficient viral spread and accumulation. The rice LRR receptor-like protein OsRLP1 is involved in the RBSDV-induced defense response by positively regulating the activation of MAPKs and PTI-related gene expression and interacting with the receptor-like kinase OsSOBIR1, which can regulate the PTI response and rice antiviral defense (Zhang et al., 2021).

Among the plant resistance-related DEGs related to 3'UTR response, seven peroxidases genes were differentially expressed in the present study, of which four were upregulated and three were downregulated. Peroxidases have been implicated in plant responses to physical stress and pathogens, as well as in a variety of cellular processes. The infection of tobacco with TMV results in the induction of two moderately anionic peroxidase isozymes in the leaves (Lagrimini and Rothstein, 1987). Sugarcane mosaic virus specifically induces the expression of the class III peroxidase gene family in sugarcane (Shang et al., 2023). Moreover, peroxidase is involved in the resistance response to pepper yellow mosaic virus (PepYMV) in *C. baccatum var. pendulum*, and increased peroxidase expression has been observed in infected plants (Gonçalves et al., 2013). One of the upregulated peroxidase genes in the present study was the glutathione peroxidase (*GPX*) gene, with log_2_ (FC) = 1.04. *GPX* is a crucial enzyme that scavenges reactive oxygen species and plays a vital role in enhancing plant stress resistance. Silencing *TaGPX3.2A* by virus-induced gene silencing led to reduced resistance of wheat to *Fusarium graminearum*, indicating the crucial role of *TaGPX3.2A* in enhancing resistance to this pathogen (Jiang et al., 2023).

Here, we only analyzed the early stage of transient 3'UTR-GFP expression of in *N. benthamiana* because 3'UTR-GFP expression was highest during this period, and DEGs were more representative. KEGG enrichment analysis showed that DEGs in the plant–pathogen interaction pathway were significantly enriched, with this pathway showing the highest enrichment level, and many genes related to plant resistance were differentially expressed. Based on this finding, we hypothesize that the 3'UTR of TVMV induces the expression of a series of resistance-related genes in plants, resulting in a decrease in GFP expression. Our results are of great significance for the study of the 3'UTR of TVMV in the mechanism of plant antiviral action and lay a foundation for research on the role of the viral non-coding region in the mechanism of plant antiviral action.

## Data Availability

The datasets presented in this study can be found in online repositories. The names of the repository/repositories and accession number(s) can be found in the article/Supplementary material.

## Footnotes

^1^ https://solgenomics.net/organism/Nicotiana_benthamiana/genome
