# Supplementary material for: 3'UTR of tobacco vein mottling virus regulates downstream GFP expression and changes in host gene expression
Source: Front Microbiol. 2024 Oct 14;15:1477074. doi: 10.3389/fmicb.2024.1477074 (PMC11514416; doi:10.3389/fmicb.2024.1477074)
Supplement: Supplementary file 7 [file Data_Sheet_7.ZIP › western blot and gel images/description of western blot and gel images.docx]

1 western blot images

Lane 1: Marker

Lane 2,3: Vector

Lane 4,5: 3'UTR-GFP

Lane 6,7: GFP

2 western blot gel images

Lane 1: Marker

Lane 2,3: Vector

Lane 4,5: 3'UTR-GFP

Lane 6,7: GFP

3 western blot template completed images

Lane 1: Marker

Lane 2,3: Vector

Lane 4,5: 3'UTR-GFP

Lane 6,7: GFP

Figure 1B

Lane 1: Marker (Sangon Biotech B600021)

Lane 2,3: 3'UTR

Figure 1C (Sangon Biotech B600021)

Lane 1: Marker

Lane 7: GFP

Figure 1D (TIANGEN 1 kb plus DNA Ladder)

Lane 1: Marker

Lane 2: 3'UTR-GFP

Figure 1E

Lane 2: Marker (Sangon Biotech B600031)

Lane 3,4: 3'UTR
