# Supplementary figures and images for: 3'UTR of tobacco vein mottling virus regulates downstream GFP expression and changes in host gene expression
Source: Front Microbiol. 2024 Oct 14;15:1477074. doi: 10.3389/fmicb.2024.1477074 (PMC11514416; doi:10.3389/fmicb.2024.1477074)

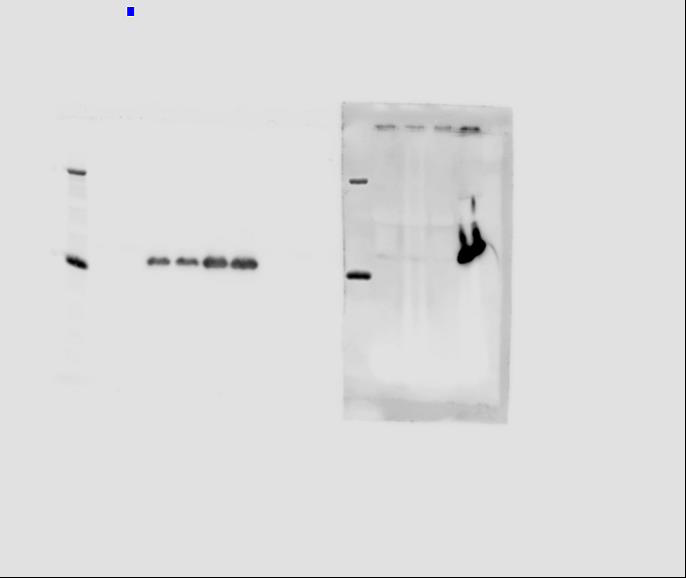

Supplement: Supplementary file 7 [file Data_Sheet_7.ZIP › western blot and gel images/1 western blot images.tif]

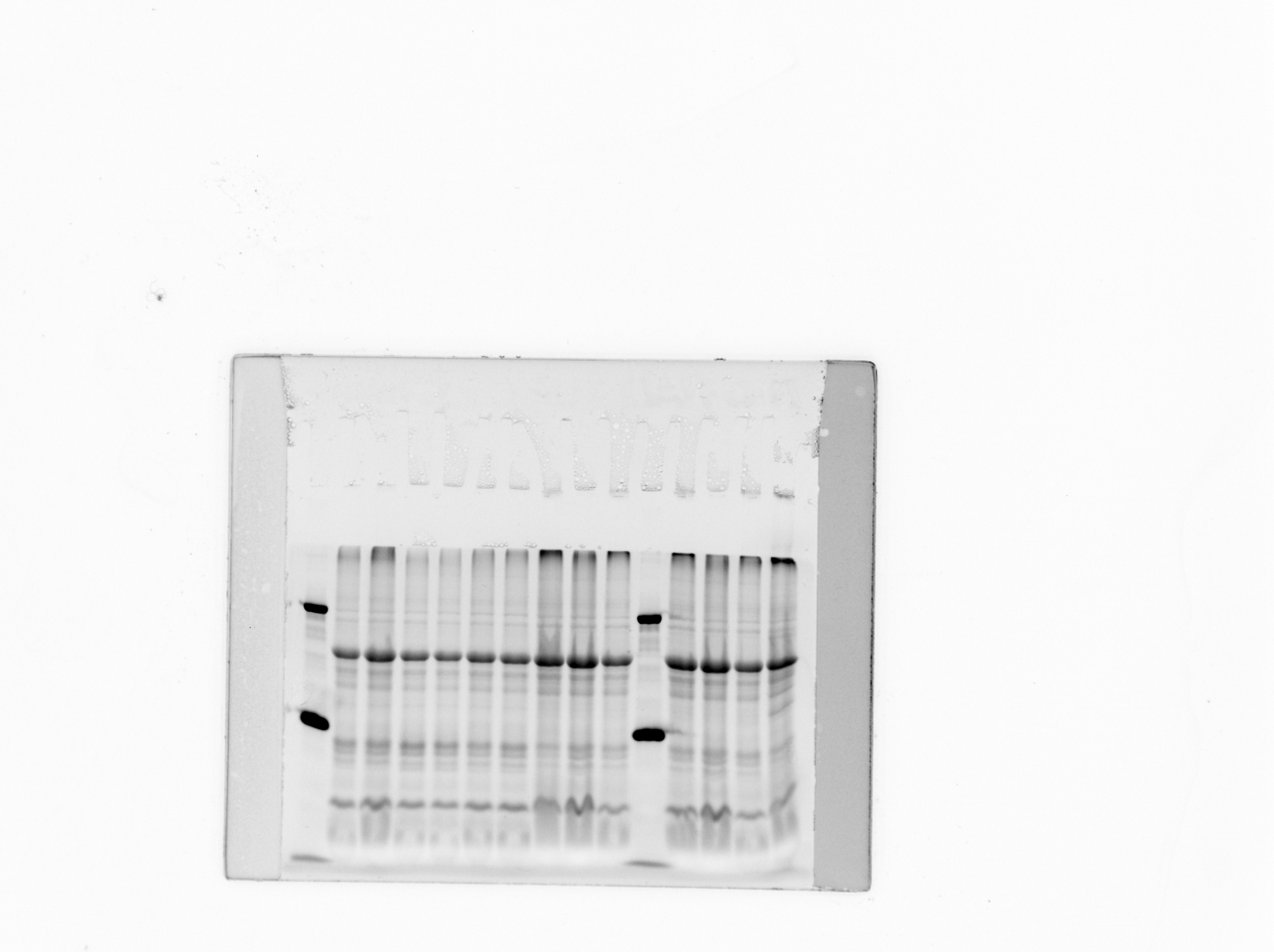

Supplement: Supplementary file 7 [file Data_Sheet_7.ZIP › western blot and gel images/2 western blot gel images.tif]

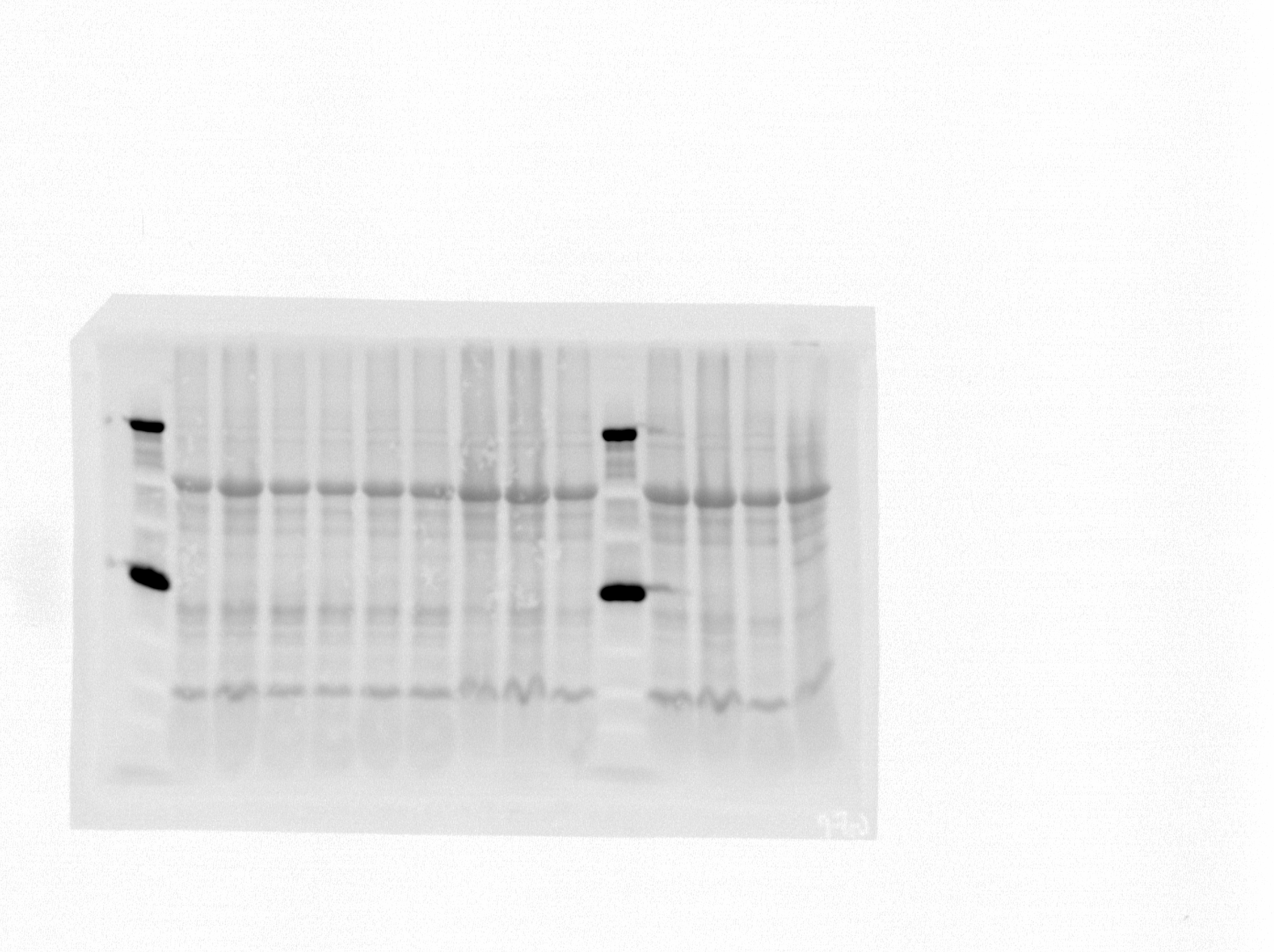

Supplement: Supplementary file 7 [file Data_Sheet_7.ZIP › western blot and gel images/3 western blot template completed images.tif]

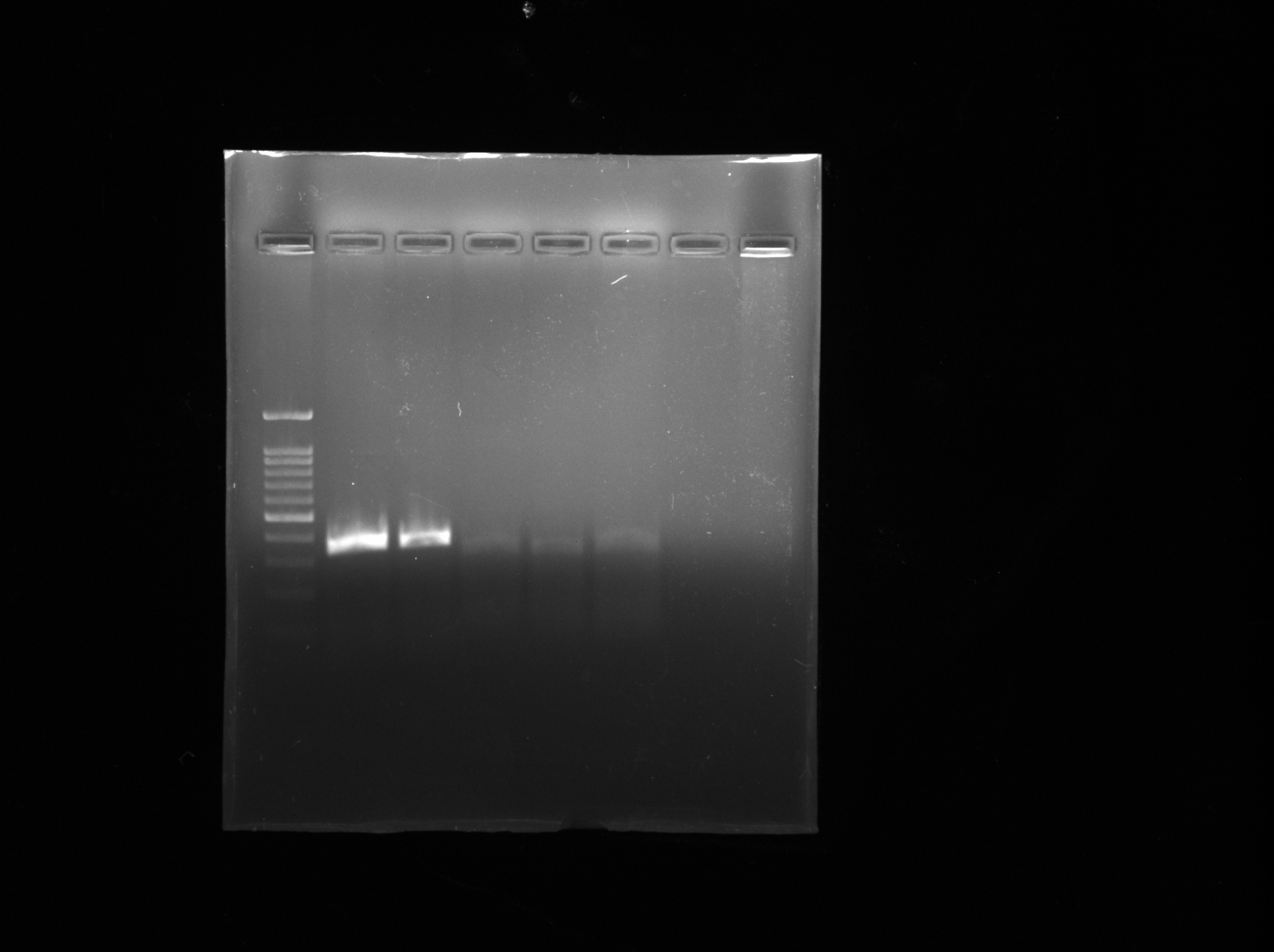

Supplement: Supplementary file 7 [file Data_Sheet_7.ZIP › western blot and gel images/Figure 1B.tif]

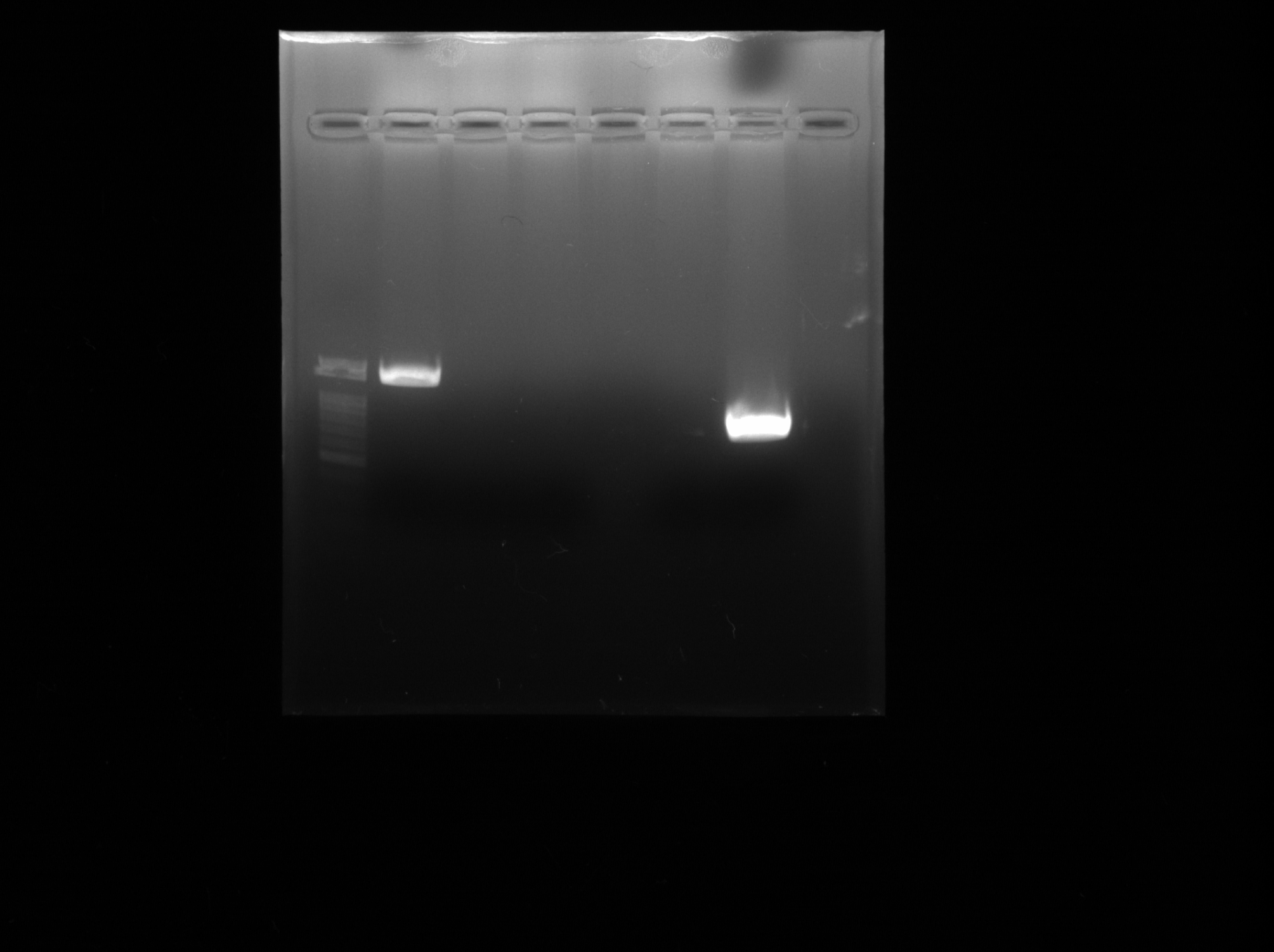

Supplement: Supplementary file 7 [file Data_Sheet_7.ZIP › western blot and gel images/Figure 1C.tif]

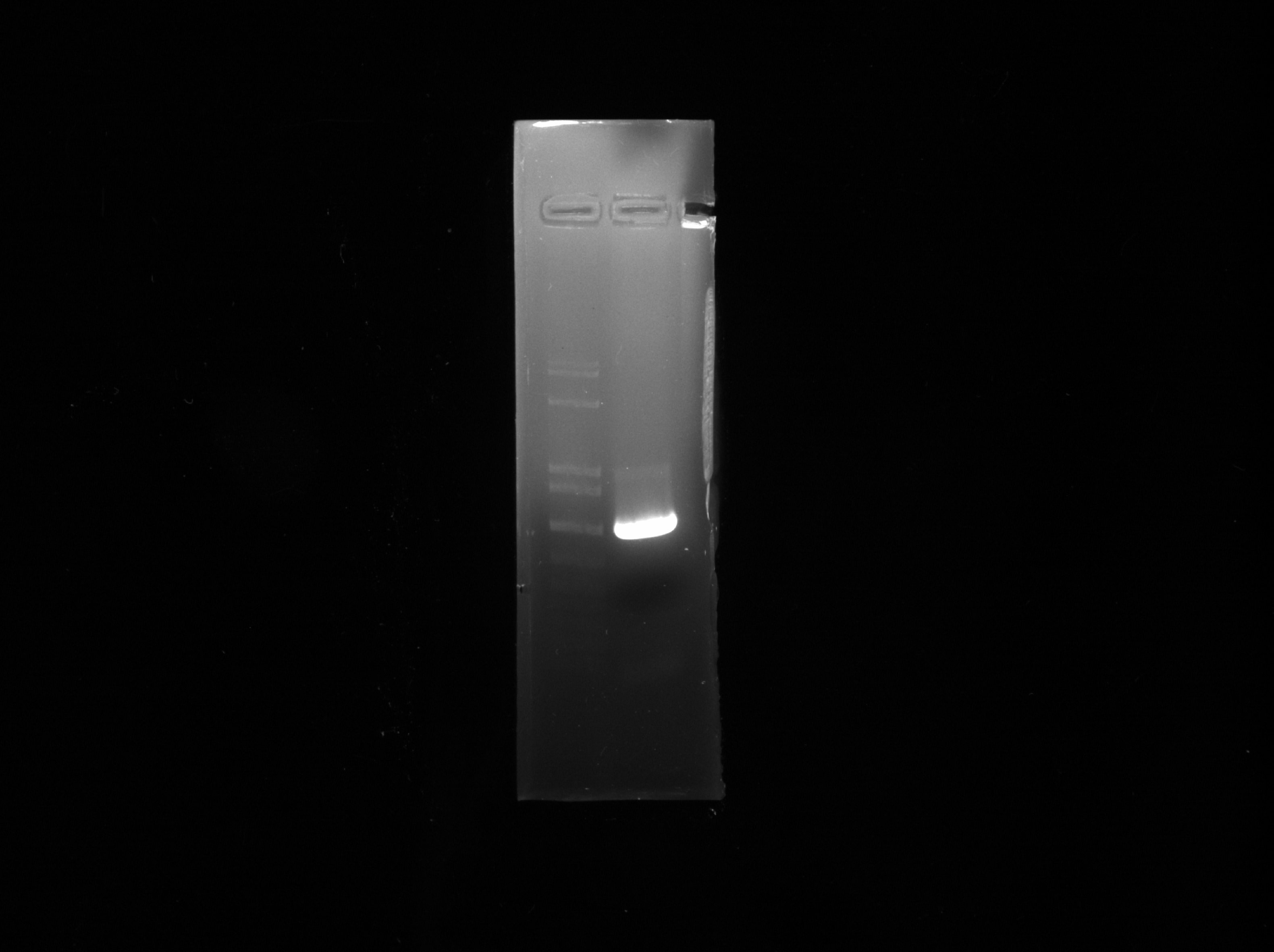

Supplement: Supplementary file 7 [file Data_Sheet_7.ZIP › western blot and gel images/Figure 1D.tif]

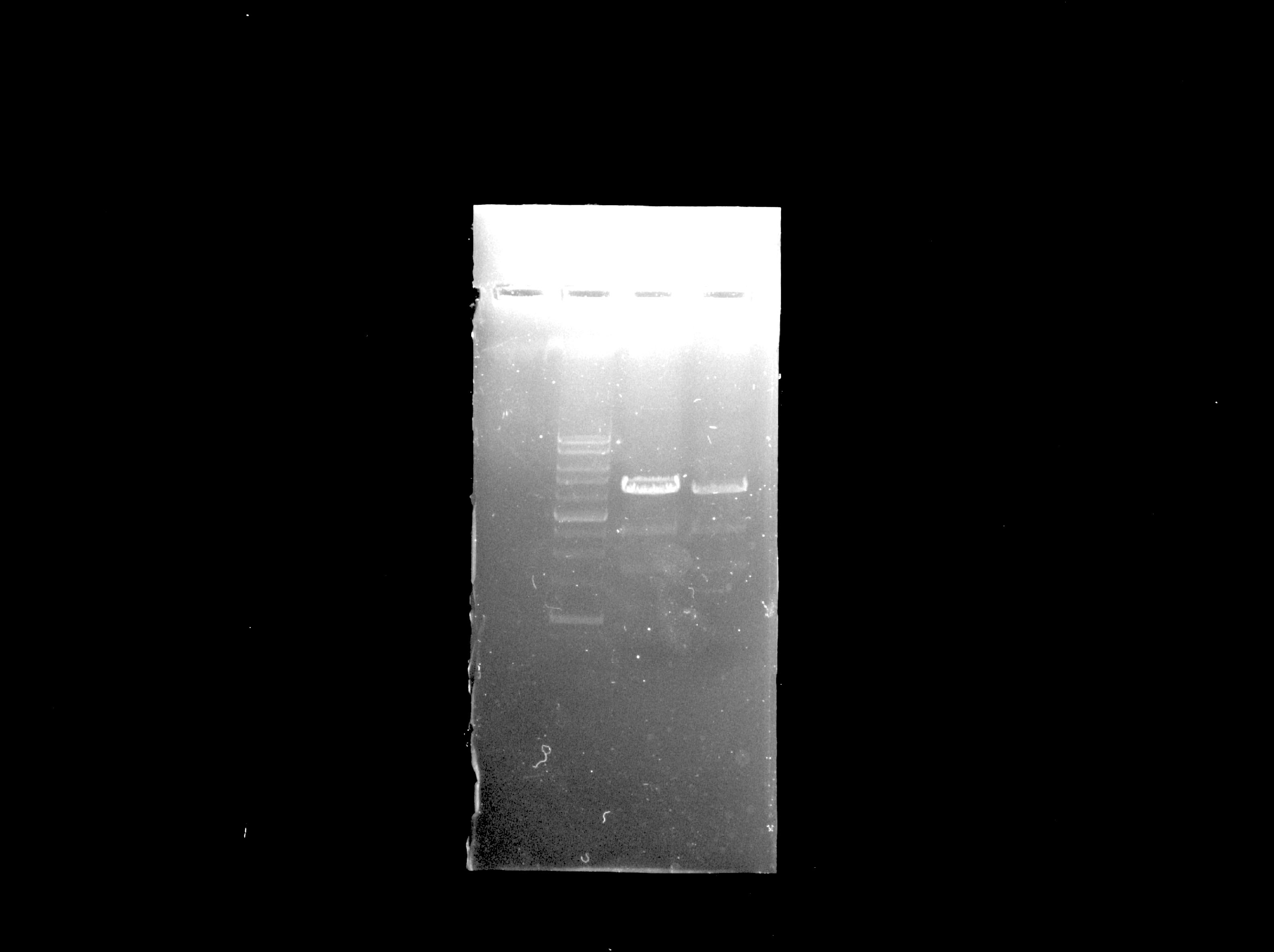

Supplement: Supplementary file 7 [file Data_Sheet_7.ZIP › western blot and gel images/Figure 1E.tif]

**Supplementary Figure 1.** Sequencing peak figures of 3'UTR-GFP in pDONR-3'UTR-GFP plasmid.


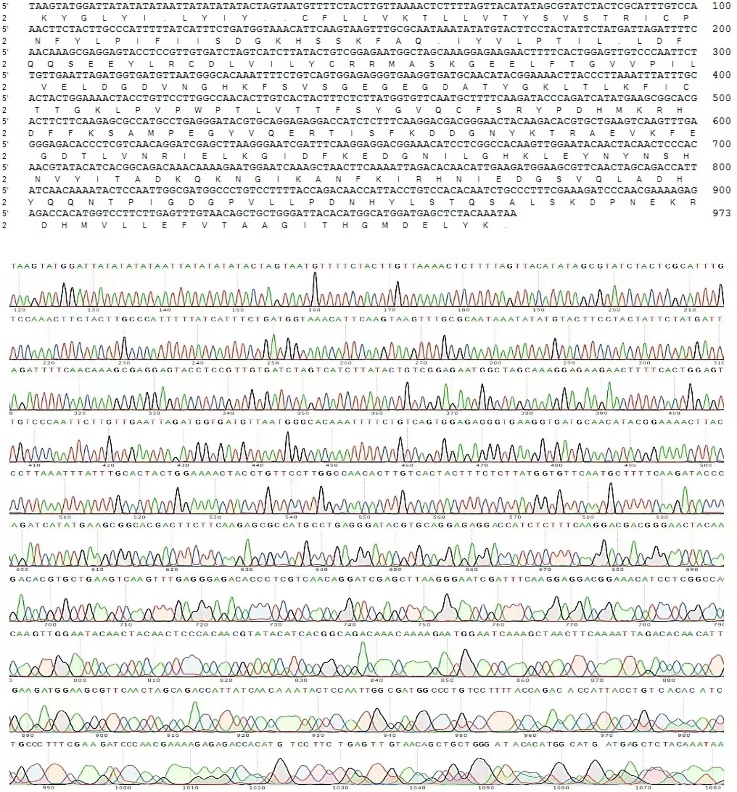

Supplement: Supplementary file 8 [file Table_1.DOCX]

**Supplementary Figure 2.** Sequencing peak figures of 3'UTR-GFP in pEAQ-3'UTR-GFP plasmid.


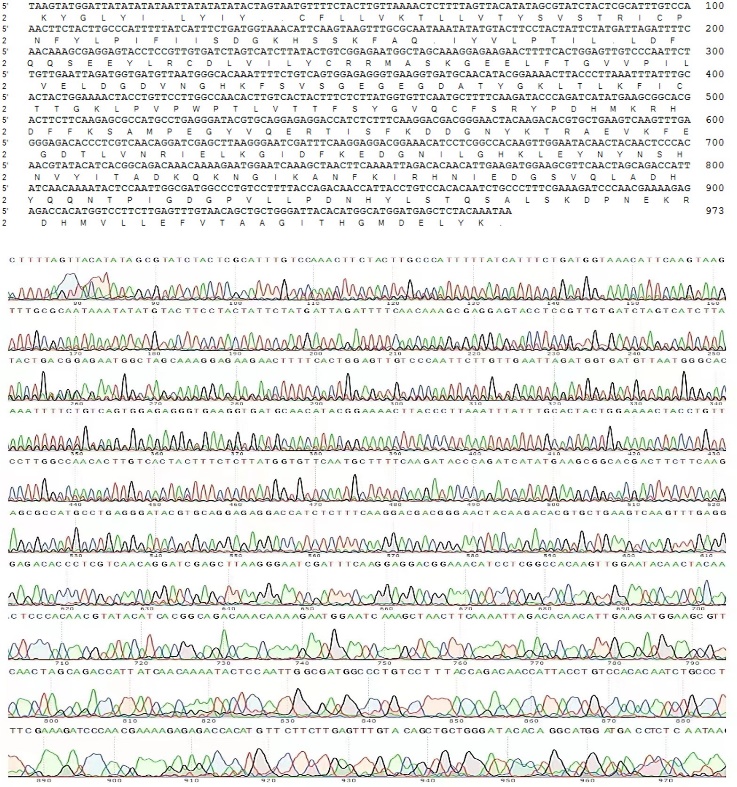

Supplement: Supplementary file 9 [file Table_2.DOCX]
