# Supplementary material for: 3'UTR of tobacco vein mottling virus regulates downstream GFP expression and changes in host gene expression
Source: Front Microbiol. 2024 Oct 14;15:1477074. doi: 10.3389/fmicb.2024.1477074 (PMC11514416; doi:10.3389/fmicb.2024.1477074)
Supplement: Supplementary file 10 [file Table_3.DOCX]

**Supplementary Figure 3.** Gene density of the cDNA library and Pearson correlation analysis of samples.


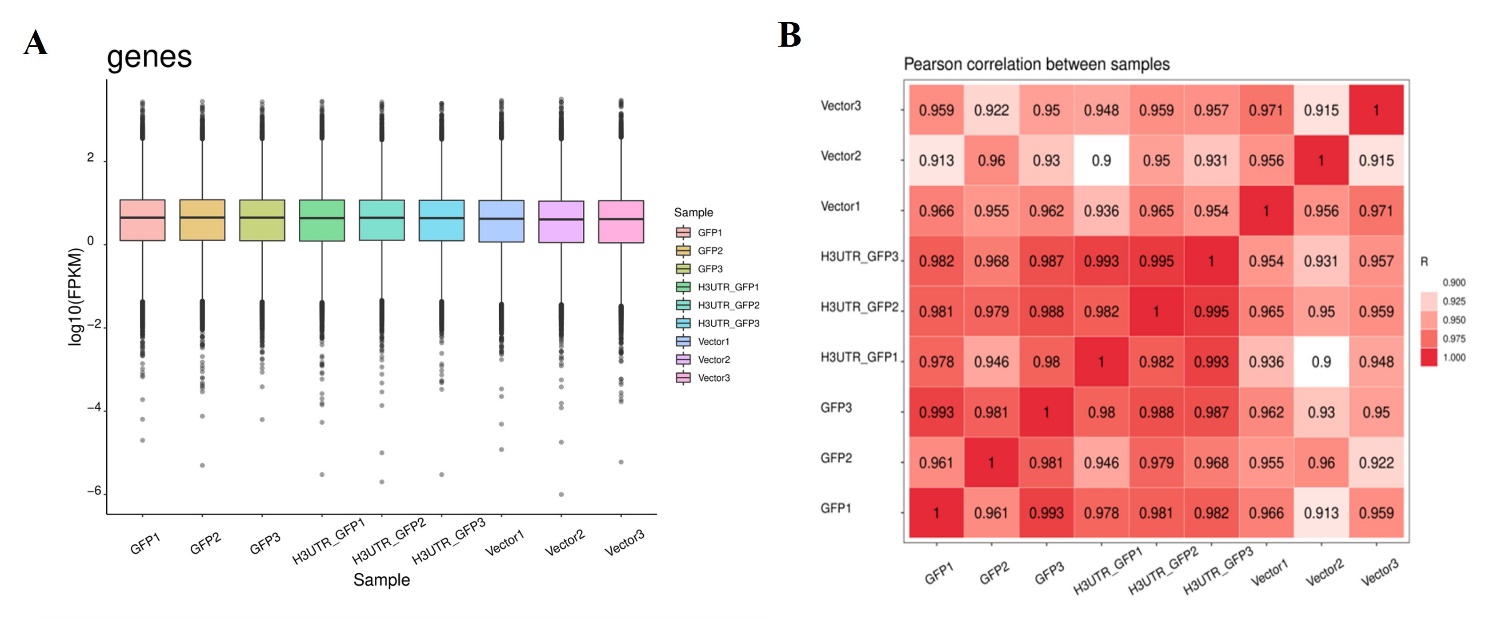


**(A)** Gene density of cDNA libraries. The abscissa is the sample name and the ordinate is log10 (FPKM); **(B)** Pearson correlation analysis of gene expression information of 3'UTR-GFP, GFP and Vector samples: Abscissa and ordinate are each sample respectively, and the color depth indicates the correlation coefficient of the two samples. The closer to the red (the closer to 1), the greater the correlation; the closer to white, the smaller the correlation.
