# Supplementary material for: 3'UTR of tobacco vein mottling virus regulates downstream GFP expression and changes in host gene expression
Source: Front Microbiol. 2024 Oct 14;15:1477074. doi: 10.3389/fmicb.2024.1477074 (PMC11514416; doi:10.3389/fmicb.2024.1477074)
Supplement: Supplementary file 11 [file Table_4.DOCX]

# Supplementary Table 1. PCR primers used in the study

| ID | Sequence (5'-3') | Size of primers (bp) | Size of expected fragments(bp) |
| --- | --- | --- | --- |
| attB-3'UTR-F1 | GGGGACAAGTTTGTACAAAAAAGCAGGCTTCTAAGTATGGATTATATATATAAT | 54 | 352 |
| 3UTR-GFP-R1 | TTCTTCTCCTTTGCTAGCCATTCTCCGACAGTATAAGATGAC | 42 |  |
| 3'UTR-GFP-F2 | GTCATCTTATACTGTCGGAGAATGGCTAGCAAAGGAGAAGAAC | 43 | 772 |
| attB-GFP-R2 | GGGGACCACTTTGTACAAGAAAGCTGGGTCTTATTTGTAGAGCTCATCCATG | 52 |  |
| GFP(el)-F | ATGGTGTTCAATGCTTTTCCCG | 22 | 129 |
| GFP(el)-R | GCGTCTTGTAGTTCCCGTCA | 20 |  |
| NtUB1-F | TCCAGGACAAGGAGGGTATCC | 21 | - |
| NtUB1-R | GTCAGCCAAGGTCCTTCCATCC | 22 |  |
| POX(el)-F | AGACTGCGTTGCCAAATCTT | 20 | 206 |
| POX(el)-R | TGCAAATCCAGCATCAATGT | 20 |  |
| WRKY6(el)-F | AGCCACACATGCACCAAATA | 20 | 244 |
| WRKY6(el)-R | TGGCCTCCCAAGATATGAAG | 20 |  |
| RK(el)-F | ATAGTTTGCGGCAGAAGGAA | 20 | 183 |
| RK(el)-R | TTGGACGCACAATAAACCAA | 20 |  |
| AE(el)-F | TAACTGCAGCTCCACAATGC | 20 | 161 |
| AE(el)-R | CGTCCACCGTATCCAAGAAT | 20 |  |
| CbFP(el)-F | AAGGCTGCGGTTGAGTTAAA | 20 | 232 |
| CbFP(el)-R | GGCAAATCATTCCTCTGCAT | 20 |  |
| ERF(el)-F | CGCCAAGGATATTCAGAAGG | 20 | 152 |
| ERF(el)-R | GCGCCTCCTCATCCATATAA | 20 |  |
| H2A(el)-F | TACGCTCAACGTGTTGGAAG | 20 | 190 |
| H2A(el)-R | TTGTCACACCAGCAAGAAGC | 20 |  |
| PP2C(el)-F | GTTACTCGGCAACGGTCAAT | 20 | 230 |
| PP2C(el)-R | CGATCCACCTCGAAATTGTT | 20 |  |
| WRKY22(el)-F | GGGAGATGAGTTCTCGTGGA | 20 | 244 |
| WRKY22(el)-R | CACTCCTTCCTCTTCCGTTG | 20 |  |
