# Supplementary material for: 3'UTR of tobacco vein mottling virus regulates downstream GFP expression and changes in host gene expression
Source: Front Microbiol. 2024 Oct 14;15:1477074. doi: 10.3389/fmicb.2024.1477074 (PMC11514416; doi:10.3389/fmicb.2024.1477074)
Supplement: Supplementary file 12 [file Table_5.DOCX]

**Supplementary Table 2.** Sequencing of 3'UTR-GFP in expression vector

| **vector** | Sequence (5'-3') |
| --- | --- |
| pDONR-3'UTR-GFP | TAAGTATGGATTATATATATAATTATATATATACTAGTAATGTTTTCTACTTGTTAAAACTCTTTTAGTTACATATAGCGTATCTACTCGCATTTGTCCAAACTTCTACTTGCCCATTTTTATCATTTCTGATGGTAAACATTCAAGTAAGTTTGCGCAATAAATATATGTACTTCCTACTATTCTATGATTAGATTTTCAACAAAGCGAGGAGTACCTCCGTTGTGATCTAGTCATCTTATACTGTCGGAGA ATGGCTAGCAAAGGAGAAGAACTTTTCACTGGAGTTGTCCCAATTCTTGTTGAATTAGATGGTGATGTTAATGGGCACAAATTTTCTGTCAGTGGAGAGGGTGAAGGTGATGCAACATACGGAAAACTTACCCTTAAATTTATTTGCACTACTGGAAAACTACCTGTTCCTTGGCCAACACTTGTCACTACTTTCTCTTATGGTGTTCAATGCTTTTCAAGATACCCAGATCATATGAAGCGGCACGACTTCTTCAAGAGCGCCATGCCTGAGGGATACGTGCAGGAGAGGACCATCTCTTTCAAGGACGACGGGAACTACAAGACACGTGCTGAAGTCAAGTTTGAGGGAGACACCCTCGTCAACAGGATCGAGCTTAAGGGAATCGATTTCAAGGAGGACGGAAACATCCTCGGCCACAAGTTGGAATACAACTACAACTCCCACAACGTATACATCACGGCAGACAAACAAAAGAATGGAATCAAAGCTAACTTCAAAATTAGACACAACATTGAAGATGGAAGCGTTCAACTAGCAGACCATTATCAACAAATACTCCAATTGGCGATGGCCCTGTCCTTTTACCAGACACCATTACCTGTCACACATCTGCCCTTTCGAAGATCCCAACGAAAAGAGAGACCACATGTCCTTCTGAGTTGTAACAGCTGCTGGGATACACATGGCATGATGAGCTCTACAAATAA |
| pEAQ-3'UTR-GFP | CTTTTAGTTACATATAGCGTATCTACTCGCATTTGTCCAAACTTCTACTTGCCCATTTTTATCATTTCTGATGGTAAACATTCAAGTAAGTTTGCGCAATAAATATATGTACTTCCTACTATTCTATGATTAGATTTTCAACAAAGCGAGGAGTACCTCCGTTGTGATCTAGTCATCTTATACTGACGGAGA ATGGCTAGCAAAGGAGAAGAACTTTTCACTGGAGTTGTCCCAATTCTTGTTGAATTAGATGGTGATGTTAATGGGCACAAATTTTCTGTCAGTGGAGAGGGTGAAGGTGATGCAACATACGGAAAACTTACCCTTAAATTTATTTGCACTACTGGAAAACTACCTGTTCCTTGGCCAACACTTGTCACTACTTTCTCTTATGGTGTTCAATGCTTTTCAAGATACCCAGATCATATGAAGCGGCACGACTTCTTCAAGAGCGCCATGCCTGAGGGATACGTGCAGGAGAGGACCATCTCTTTCAAGGACGACGGGAACTACAAGACACGTGCTGAAGTCAAGTTTGAGGGAGACACCCTCGTCAACAGGATCGAGCTTAAGGGAATCGATTTCAAGGAGGACGGAAACATCCTCGGCCACAAGTTGGAATACAACTACAACTCCCACAACGTATACATCACGGCAGACAAACAAAAGAATGGAATCAAAGCTAACTTCAAAATTAGACACAACATTGAAGATGGAAGCGTTCAACTAGCAGACCATTATCAACAAAATACTCCAATTGGCGATGGCCCTGTCCTTTACCAGACAACCATTACCTGTCCACACAATCTGCCCTTTCGAAAGATCCCAACGAAAAGAGAGACCACATGTTCTTCTTGAGTTTGTACAGCTGCTGGGATACACAGGCATGGATGACCTCTCAATAA |
