# Supplementary material for: 3'UTR of tobacco vein mottling virus regulates downstream GFP expression and changes in host gene expression
Source: Front Microbiol. 2024 Oct 14;15:1477074. doi: 10.3389/fmicb.2024.1477074 (PMC11514416; doi:10.3389/fmicb.2024.1477074)
Supplement: Supplementary file 13 [file Table_6.DOCX]

**Supplementary Table 3.** RNA-Seq data and sequence comparison results of sample sequencing data with the reference genome

| Samples name | Raw reads | Clean reads | Clean bases | Q20% | Q30% | GC content % | Mapped reads | Uniq mapped reads | Multi mapped reads |
| --- | --- | --- | --- | --- | --- | --- | --- | --- | --- |
| 3UTR-GFP-1 | 46941228 | 35954886 | 5.39G | 99.98% | 98.20% | 42.5% | 33121206 (92.12%) | 30760845 (85.55%) | 2360361 (6.56%) |
| 3UTR-GFP-2 | 44513864 | 33927468 | 5.09G | 99.97% | 98.22% | 42.5% | 31306770 (92.28%) | 29040452 (85.60%) | 2266318 (6.68%) |
| 3UTR-GFP-3 | 43923106 | 33663078 | 5.05G | 99.98% | 98.28% | 42.5% | 30987717 (92.05%) | 28762959 (85.44%) | 2224758 (6.61%) |
| GFP-1 | 50973536 | 35271336 | 5.29G | 99.97% | 98.24% | 42.5% | 32586509 (92.39%) | 30289771 (85.88%) | 2296738 (6.51%) |
| GFP-2 | 53830988 | 35229754 | 5.28G | 99.98% | 98.25% | 42.5% | 32420055 (92.02%) | 30129864 (85.52%) | 2290191 (6.50%) |
| GFP-3 | 54401868 | 36742398 | 5.51G | 99.97% | 98.23% | 42.5% | 33636713 (91.55%) | 31276357 (85.12%) | 2360356 (6.42%) |
| Vector-1 | 42763280 | 39583272 | 5.94G | 99.98% | 98.25% | 42.5% | 35476885 (89.63%) | 32868654 (83.04%) | 2608231 (6.59%) |
| Vector-2 | 44611518 | 41908992 | 6.29G | 99.97% | 98.25% | 42.5% | 38458421 (91.77%) | 35557642 (84.84%) | 2900779 (6.92%) |
| Vector-3 | 45460206 | 42170250 | 6.33G | 99.98% | 98.25% | 42.5% | 38173017 (90.52%) | 35294081 (83.69%) | 2878936 (6.83%) |
